# Carbon pathways and trophic attributes are conserved in carnivorous reef fishes across a major human disturbance gradient

**DOI:** 10.1111/1365-2656.70151

**Published:** 2025-10-12

**Authors:** Matthew D. Ramirez, Kelton W. McMahon, Neil Rooney, Rana W. El‐Sabaawi, Julia K. Baum

**Affiliations:** ^1^ Department of Biology University of Victoria Victoria British Columbia Canada; ^2^ Graduate School of Oceanography University of Rhode Island Narragansett Rhode Island USA; ^3^ Department of Biology and Marine Biology University of North Carolina Wilmington Wilmington North Carolina USA; ^4^ Center for Marine Science University of North Carolina Wilmington Wilmington North Carolina USA; ^5^ School of Environmental Sciences University of Guelph Guelph Ontario Canada; ^6^ Hawaii Institute of Marine Biology University of Hawaii Kaneohe Hawaii USA

**Keywords:** amino acids, carbon source, compound‐specific stable isotope analysis, coral reefs, food web, human disturbance, isotopic niche, trophic position

## Abstract

1. Habitat degradation and overexploitation are key drivers of biodiversity loss globally. Negative, human‐driven changes in habitat quality, species abundance, and community composition are well‐documented across systems. While it is understood that human stressors destabilize consumer‐resource interactions, how energy pathways and food webs may reorganize in hyperdiverse tropical systems in response to human disturbance remains poorly understood due to their complexity and past methodological limitations.
2. Leveraging recent advances in molecular isotope techniques, we performed an ecosystem‐scale natural experiment to evaluate how human disturbance reorganizes carbon pathways and trophic structure in a hyperdiverse tropical system, Kiritimati Island, the world's largest atoll. We specifically employed novel integrations of bulk tissue and amino acid‐specific stable isotope analyses applied to six nominally generalist fish species sampled across Kiritimati's well‐documented human disturbance gradient. Sampled fish species comprised 48% of carnivorous reef fish biomass.
3. Essential amino acid stable carbon isotope (δ^13^C_EAA_) fingerprinting and Bayesian stable isotope mixing models indicated that the proportional contribution of the carbon sources supporting five of the six sampled fish species did not vary across the disturbance gradient. Energy disproportionately (>80%) originated from planktonic production and microbially reworked detritus for most species, with only minor contributions of carbon sourced from coral and epilithic algal matrices. Reef fish trophic ecology was also consistent across the atoll, with species maintaining isotopic niches (size and position) and trophic positions across the atoll despite significant human disturbance‐mediated changes in community composition and habitat complexity.
4. Our findings suggest that the energy channels connecting basal resources to upper trophic level generalist consumers can be highly conserved following significant human disturbance in hyperdiverse tropical systems. On coral atolls, specifically, robust planktonic or detrital energy channels may buffer nominally generalist carnivorous reef fishes from some negative effects of chronic local human disturbance, promoting the maintenance of dominant energy fluxes in disturbed habitats. These results illustrate that disturbance‐mediated changes in ecosystem structure and function do not universally destabilize broad energy fluxes and trophic relationships in hyperdiverse ecosystems. On the contrary, there appear to be mechanisms that promote stability, such as broad reliance on system‐dominant production sources.

## Graphical abstract

Our research leverages a natural, ecosystem‐scale experiment and cutting‐edge molecular isotope approaches to reveal that coral reef food web structure and energy flow can remain consistent across a gradient of human disturbance.

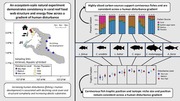

## 1 INTRODUCTION

Biodiversity, food web structure and ecosystem function and stability are inextricably linked (Rooney & McCann, 2012; Thompson et al., 2012), with diverse systems tending to be more stable in changing environments (Loreau & De Mazancourt, 2013; Tilman et al., 2006). Theory predicts that adaptive food web structures, such as diverse energy channels coupled by mobile consumers, promote food web stability (Rooney et al., 2006). Human‐driven biodiversity loss caused by climate change and other long‐standing anthropogenic stressors (e.g. overfishing, development, pollution) is known to destabilize consumer‐resource interactions (IPCC, 2022; Valiente‐Banuet et al., 2015), eroding the structures and processes that promote food web stability (O'Gorman et al., 2019). Asymmetric species responses can rewire both food web topology (who eats whom) and interaction strengths (O'Gorman & Emmerson, 2009), often through behavioural responses of mobile generalist consumers to physiological constraints imposed by new environmental conditions and changes in resource availability or distribution (Bartley et al., 2019; Rahman & Candolin, 2022). These changes are expected to alter food web properties that underpin the system's stability (Rooney et al., 2006), including carbon source contributions, food chain length, and ecological niche breadth. As a result, a better understanding of how human disturbance ‘rewires’ ecological networks is needed, including the identification of food web attributes most sensitive to anthropogenic changes and underlying mechanisms that may promote ecosystem stability (Lewis, 2009). In particular, there is a need to study disturbance‐mediated changes in hyperdiverse tropical systems given that they contain a disproportionate amount of Earth's biodiversity (>75% of known species) and are experiencing among the highest rates of environmental, socio‐economic and demographic change globally (Barlow et al., 2018).

Documenting networks of interactions is a time‐consuming task that is even more challenging in hyperdiverse systems because of their complexity, which has impeded our understanding of their dynamics in the face of disturbance. Although it is well established that tropical forest land use change reduces species and functional richness (Giam et al., 2015; Wilkinson et al., 2018), food web responses are often more variable and less well studied. For example, intensive agricultural practices can reduce and simplify energy flow in tropical forest terrestrial systems (Barnes et al., 2014; Tylianakis et al., 2007), but do not necessarily alter contributions of allochthonous versus autochthonous carbon to streams (Wilkinson et al., 2021). Effects of land use change on food chain length in tropical forest streams are mixed (Chua et al., 2021; Wilkinson et al., 2021). Similarly, although we know that human‐driven coral losses and shifts in coral reef community composition can disrupt reef fish trophic structure and shorten food chain length (Hempson, Graham, MacNeil, Bodin, et al., 2018; Hempson, Graham, MacNeil, Hoey, et al., 2018), whether such changes are associated with altered energy fluxes from basal resources to predators has yet to be evaluated. Recent research suggests that coral reef trophic pathways are maintained by dietary and habitat specialists (McMahon et al., 2025; Pozas‐Schacre et al., 2021), species whose persistence is vulnerable to loss of structural complexity (Rogers et al., 2014; Wilson et al., 2006). As a result, human‐driven coral reef degradation may strongly influence trophic structure and energy fluxes through effects on dietary specialists. Mobile generalist consumers that behaviourally couple diverse energy channels may provide a mechanism that confers stability on disturbed coral reef food webs (Rooney et al., 2006).

Characterizing energy flow through hyperdiverse food webs has also been particularly challenging due to limitations of widely applied tools. Bulk tissue stable isotope analyses have garnered widespread use in food web ecology but generally cannot be used to precisely characterize energy fluxes due to high overlap in bulk tissue stable isotope values among isotope source pools and between producers and consumers (Layman et al., 2012). Recently, compound‐specific stable isotope analysis of amino acids (CSIA‐AA) has transformed our ability to precisely characterize the origin and flux of energy within ecosystems by unlocking unique metabolic information contained within individual amino acids. Diversity in essential amino acid biosynthesis pathways within basal production sources yields taxonomically diagnostic stable carbon isotope (δ^13^C_EAA_) ‘fingerprints’ that can be used to identify diverse basal production sources fueling the food webs supporting upper trophic level consumers (Larsen et al., 2013; McMahon et al., 2010). Additionally, the stable nitrogen isotope (δ^15^N) values of heavily fractionating ‘trophic’ amino acids and minimally fractionating ‘source’ amino acids (McMahon & McCarthy, 2016) provide consumer trophic position estimates that are internally indexed to variations in ecosystem nitrogen isotope biogeochemical cycling (Chikaraishi et al., 2009). CSIA‐AA has already provided important insights on the production sources fueling coral reefs (McMahon et al., 2016; Skinner et al., 2021) as well as consumer trophic ecology (Bradley et al., 2016; Papastamatiou et al., 2015) via single‐isotope studies. However, few studies have applied these tools synergistically to characterize stressor impacts on food web architecture (but see Fujii et al., 2020; McMahon et al., 2025; Wilkinson et al., 2021).

Here, we leveraged a natural, ecosystem‐scale experiment coupled with cutting‐edge molecular isotope techniques to advance our understanding of how human disturbance reshapes energy pathways within coral reef food webs. We specifically used novel integrations of bulk tissue and molecular stable isotope approaches applied to six nominally generalist and socio‐culturally important carnivorous reef fishes to characterize variation in baseline energy source contributions, isotopic niche width and position, and trophic position across the world's largest atoll (Kiritimati, central equatorial Pacific Ocean). These six species (two generalist carnivores, four piscivores) together comprised almost half (48%) of the reef's total carnivorous fish biomass (44% of the generalist carnivore biomass, 50% of the piscivore biomass) (derived from Magel et al., 2020). Kiritimati has a well‐documented human disturbance gradient that, at the time of this study, spanned isolated, near‐pristine reefs to heavily degraded reefs that had experienced significant ecosystem disturbances (e.g. loss of reef‐building scleractinian (stony) corals, changes in fish community composition) due to highly localized fishing pressure and human development (Baum et al., 2023; Walsh, 2011; Watson et al., 2016). We hypothesized that disturbance‐mediated changes in habitat quality and community composition are associated with an expansion of carnivore trophic niches and a decline in food chain length (e.g. reduced functional diversity), which would manifest as declines in carnivore trophic positions. We also hypothesized that human disturbance reconfigures energy flow pathways, shifting the system from one supported by diverse baseline energy sources (e.g. coral, epilithic algal matrix, detritus, plankton; very low disturbance) to one supported by fewer taxa (e.g. epilithic algal matrix, plankton; very high disturbance).

## 2 MATERIALS AND METHODS

### 2.1 Study location and sample collection

Kiritimati (Christmas Island), Republic of Kiribati, is located in the central equatorial Pacific Ocean (01°52′ N, 157°24′ W) and is the world's largest atoll by landmass (388 km^2^, 150 km in perimeter). Kiritimati has a well‐documented human disturbance gradient that includes very high disturbance sites that are situated near population centres and affected by relatively high localized coastal development (dredging, infrastructure, pollution) and subsistence fishing, medium disturbance sites that are affected by moderate fishing pressure only, and sites with very low disturbance that contain near‐pristine reefs spatially isolated from most human activities at the time of this study (Table 1). Very low disturbance sites were dominated by hard coral cover (52.4%; Baum et al., 2023). In contrast, very high disturbance sites had very low hard coral cover (12.2%), with most of the benthic community composed of turf algae (31.3%) and abiotic substrates (45.4%; sediment, sand, rubble). Surface rugosity and terrain ruggedness were inversely related to disturbance level (Magel et al., 2019), and fish community composition varied across sites (Magel et al., 2020). The oceanography around the atoll is broadly controlled by the Southeast Trade Winds that propel the South Equatorial Current westward (Walsh, 2011), which creates an island‐wake upwelling zone along the broad lagoon mouth on its northwestern side of the atoll with relatively high nutrient concentrations (DeMartini et al., 2008).

**TABLE 1:** Variability in human disturbance and benthic cover across sampling sites on Kiritimati atoll.

Site	Human population	Fishing pressure	Combined metric	Percent cover (%)						
				Hard coral	Soft coral	CCA	Turf	Macro	Abiotic	Other
VH1^a^, ^b^	4042	3234	7276	1.7	0.0	0.2	28.5	7.7	59.3	2.6
VH3^a^, ^b^	3065	2021	5086	32.6	3.1	6.7	13.8	1.5	41.7	0.7
M1^a^, ^b^	0	1213	1213	35.3	1.5	8.3	10.5	6.0	37.5	0.9
M2^a^, ^b^	0	1213	1213	47.2	2.6	7.5	10.0	4.3	28.0	0.3
M3^a^, ^b^	0	1213	1213	43.0	2.2	6.3	15.6	6.5	26.2	0.1
M4^a^	351	809	1160	25.7	1.3	1.9	12.4	4.8	53.5	0.4
VL1^a^, ^b^	0	0	0	61.0	0.0	13.9	22.4	0.0	2.6	0.1
VL2^a^, ^b^	0	0	0	58.9	1.0	19.9	18.4	0.4	1.3	0.1
VL6^a^, ^b^	0	0	0	n.d.	n.d.	n.d.	n.d.	n.d.	n.d.	n.d.
VL9^b^	0	0	0	n.d.	n.d.	n.d.	n.d.	n.d.	n.d.	n.d.
VL11^a^, ^b^	0	0	0	n.d.	n.d.	n.d.	n.d.	n.d.	n.d.	n.d.
			Average (SD)	38.2 (19.2)	1.5 (1.1)	8.1 (6.3)	16.4 (6.4)	3.9 (2.9)	31.3 (21.3)	0.7 (0.9)
			Range	1.7–61.0	0.0–3.1	0.2–19.9	10.0–28.5	0.0–7.7	1.3–59.3	0.1–2.6

*Note*: Human population is the number of people residing within 2 km of the site as reported by the Government of Kiribati's 2015 population census (Morate, 2016). Fishing pressure is the extracted value from a kernel density function of fishing pressure (Watson et al., 2016). Combined metric is the sum of population and fishing pressure, and sites are ordered from greatest to least disturbance according to this metric. Percent benthic cover prior to the 2015–2016 El Niño reproduced from Baum et al. (2023).

Abbreviations: Abiotic, sediment, sand, and rubble; CCA, crustose coralline algae; M, medium; Macro, macroalgae; Turf, benthic turf algae; VH, very high; VL, very low.

^a^ Fish sampled for bulk tissue stable isotope analysis.

^b^ Fish sampled for compound‐specific stable isotope analysis.

**FIGURE 1:**
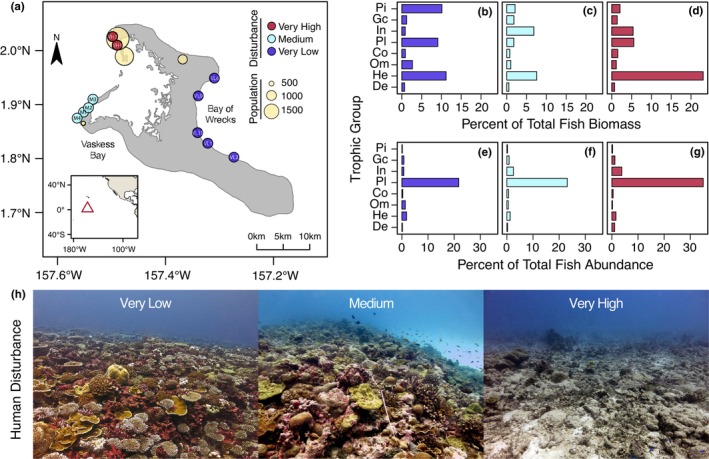
(a) Map of Kiritimati atoll (Christmas Island; central equatorial Pacific Ocean) noting sampling locations and designated levels of human disturbance, quantified as a function of human population density and subsistence fishing pressure (Table 1). Relative biomass (b–d) and abundance (e–g) of fish trophic groups by disturbance level quantified via underwater visual censuses of the reef fish communities (modified from Magel et al., 2020); Pi = piscivore, GC = generalist carnivore, De = detritivore, In = invertivore, Pl = planktivore, Co = corallivore, Om = omnivore, He = herbivore. (H) Photos of the coral reef communities at sites representing each of the atoll's levels of human disturbance.

In July–August of 2010–2012, scientific divers used spearfishing to collect specimens of six of the most abundant and socio‐culturally important carnivorous reef fish species at 11 sites (8–12 m depth) across Kiritimati's forereefs (Figure 1; generalist carnivores: blacktail snapper, *Lutjanus fulvus*; darkfin hind, *Cephalopholis urodeta*; piscivores: two‐spot red snapper, *Lutjanus bohar*; peacock hind, *Cephalopholis argus*; bluefin trevally, *Caranx melampygus*; small toothed jobfish, *Aphareus furca*). Fishes within the Lutjanidae and Carangidae families are among the most commonly caught fish species in Kiritimati (Watson et al., 2016). Critically, this sampling took place before the 3rd global coral bleaching event, which was driven by the 2015–2016 El Niño that caused an almost 90% loss of hard coral cover on the atoll (Baum et al., 2023), with unknown consequences for the reef food web. Fish were stored on ice and dissected the evening they were caught. Prior to dissection, each specimen was photographed, weighed, and measured to the nearest millimetre with vernier callipers for standard and total length (Table S1). We excised a small sample (~10 g) of dorso‐lateral white muscle tissue from each fish that was then stored frozen until further processing. At the University of Victoria, each muscle sample was rinsed with deionized water, dried at 60°C for 48 h, and homogenized with a mortar and pestle. Fish collection was performed collaboratively with scientists at multiple institutions, and methods were approved by Institutional Animal Care and Use Committees at the University of Victoria, Simon Fraser University, and Stanford University. Annual scientific research permits were issued by the Environment and Conservation Division within the Republic of Kiribati's Ministry of Environment, Lands and Agriculture Development.

Each species has variable mobility and habitat associations but spatial home ranges that generally constrain them to sampling sites within disturbance areas, which are >10 km apart (Figure 1; see Supporting Information). *L. fulvus*, *C. urodeta*, *L. bohar*, *C. argus* and *C. melampygus* are strongly reef‐associated for foraging. Tracking studies are lacking for most of these species, but studies of other *Cephalopholis* and *Lutjanus* sp. suggest that hind and snapper have high site fidelity and narrow home ranges (0.1–3.6 km^2^; e.g., Liu & Sadovy, 2005; Nanami & Yamada, 2008). *C. melampygus* generally has home ranges <1 km^2^ (Holland et al., 1996). *A. furca* is the most transient of the species sampled and uses both benthic and pelagic habitats (Mundy, 2005), although home range size remains unknown. Additionally, medium and very high disturbance sites are separated by a channel with minimal coral cover, which acts as a hard migration barrier for many adult coral reef fishes (McMahon et al., 2012).

We also collected specimens of four specialized primary consumers (*n* = 5 per disturbance level per species; 60 total fish) to serve as carbon source proxies for δ^13^C_EAA_ fingerprinting (McMahon et al., 2016), a common and well‐validated approach used in lieu of in situ end member sampling within CSIA‐AA food web studies (see Supporting Information). Coral, detritus, benthic turf algae (within the epilithic algal matrix) and phytoplankton are the dominant carbon sources on Kiritimati (Table 1). Carbon source proxies, chosen based on their distinct obligate feeding ecology and ubiquity on Kiritimati (see Supporting Information), included an obligate corallivore (coral: *Chaetodon ornatissimus*), detritivore (detritus: *Ctenochaetus marginatus*), herbivore (epilithic algal matrix; *Acanthurus nigricans*) and planktivore (phytoplankton: *Pseudanthias olivaceus*). *C. ornatissimus* feeds strictly on coral polyps or mucus (Harmelin‐Vivien & Bouchon‐Navaro, 1983). *C. marginatus* feeds primarily on detritus and sediment (Randall, 2005). *A. nigricans* is an herbivorous grazer that feeds solely on benthic turf algae on Kiritimati (Kindinger et al., 2024). *P. olivaceus* is a diurnal zooplanktivore (Randall, 2005). The epilithic algal matrix includes benthic turf algae, non‐living organic components (detritus), microbial components, and inorganic material (sediment) (Wilson & Bellwood, 1997). We did not sample a fish proxy for upright, fleshy macroalgae because macroalgae is uncommon on Kiritimati (Table 1). An inherent limitation to this carbon source proxy approach is that fish proxies cannot possibly integrate all possible species within a production class. Nevertheless, this approach has a number of benefits over in situ sampling of production sources, including that among‐group isotope fingerprint differences are far larger than within group differences (Fox et al., 2019; McMahon et al., 2016; Skinner et al., 2021; Tietbohl, 2016), it avoids mismatches in isotope turnover rate of primary producers vs. consumers (Whiteman et al., 2019), and reflects only assimilated isotope signals (see Supporting Information). There are often also logistical (e.g. detritus) and ethical (e.g. CITES restricted organisms like corals) challenges associated with collecting production sources.

### 2.2 Human disturbance metric

As in Claar et al. (2020) and Magel et al. (2020), we quantified local human disturbance at our surveyed sites as a function of (1) human population density, defined as the number of people residing within 2 km of each sampling site as reported by the Government of Kiribati's 2015 population census (Morate, 2016), and (2) subsistence fishing pressure, quantified through detailed semi‐structured interviews in 2013 and summarized as a kernel density function (Watson et al., 2016). We combined these data with equal weight (population density + fishing pressure) to create a continuous, quantitative metric of chronic local human disturbance at each sampling site. In statistical analyses, human disturbance was typically treated as a continuous variable, rescaled using the *arm* package in R to account for large gaps in values among disturbance levels. For visualization purposes, human disturbance level was treated as a categorical variable (very low, medium, very high), based on clear breakpoints in the continuous disturbance metric (Table 1) (Claar et al., 2020; Magel et al., 2020).

### 2.3 Bulk tissue stable isotope analysis: Isotopic niche

We employed bulk tissue δ^13^C and δ^15^N analyses to quantify and compare within‐species isotopic niche widths and overlap among human disturbance levels. An organism's isotopic niche reflects the isotopic variation encompassed in their resource use and is thus often used as a proxy for ecological niche width (Newsome et al., 2007). A total of 232 carnivorous reef fishes were sampled (average *n* = 13 per species per disturbance level; see Table S1). Approximately, ~1.0 mg of ground muscle tissue was analysed via elemental analyser‐isotope ratio mass spectrometry (EA‐IRMS) at the University of Victoria, British Columbia, Canada (Thermo Scientific Delta IV IR‐MS) or the University of Windsor, Ontario, Canada (Thermo Scientific Delta V IR‐MS). Analytical precision was measured using four standards (internal standard tilapia, NIST bovine liver, USGS 40, IVA33802174 UREA) and was 0.09–0.13‰ for δ^13^C and 0.13–0.18‰ for δ^15^N. C:N ratios (%C divided by %N) were below 3.5, characteristic of unaltered protein with low lipid content (Post et al., 2007). Various fish samples (*n* = 141) were analysed in both laboratories for this and other studies. Resulting δ^13^C and δ^15^N values differed by an average of 0.5 and 0.4‰, respectively, which is not ecologically significant in the context of analytical uncertainty and observed within‐species variation herein. Prior to isotopic niche analyses, bulk tissue δ^13^C and δ^15^N data were baseline‐corrected using mean species‐ and site‐specific essential amino acid (δ^13^C) and source amino acid (δ^15^N) stable isotope data generated in this study (see Supporting Information).

To characterize variation in isotopic niche width across the human disturbance gradient, we quantified standard ellipse area (SEA) for each species‐disturbance level combination using both frequentist (SEAc, sample size corrected; *c*. 40% of the data) and Bayesian approaches (SEA‐B) using *SIBER* (Jackson et al., 2011) (see Supporting Information). For each species, to test for differences in isotopic niche size between disturbance levels we compared each pair of SEA‐B posterior draws for each set of disturbance levels (e.g. very high–medium, etc.) and quantified the proportion that were smaller (or larger) in magnitude. Differences in SEA‐B were considered significant if ≥95% of posterior draws were smaller or larger than the other. To quantify how a species' isotopic niche position varies across the human disturbance gradient, we quantified the percent probability of isotopic niche space overlap among disturbance levels for each species using *nicheROVER* (Swanson et al., 2015) in R. Here, the approach uses Markov chain Monte Carlo simulation to calculate the probability that, for a given species, an individual from one disturbance level occurs within the 95% isotopic niche region of another disturbance level.

### 2.4 Compound‐specific stable isotope analysis: carbon source contribution and trophic position

We employed amino acid‐specific δ^13^C and δ^15^N analyses to quantify the proportional contribution of carbon sources to carnivorous fish food webs (AA δ^13^C; *n* = 60 carbon source proxy fishes + 84 carnivorous reef fishes) and consumer trophic position (AA δ^15^N; *n* = 84 carnivorous reef fishes). For 144 total fish sampled for bulk tissue stable isotope analysis (average *n* = 5 per species per disturbance level; see Table S1), we analysed muscle samples (~5–7 mg) via CSIA‐AA at the University of Rhode Island, Narragansett, Rhode Island, USA, following protocols modified from McMahon et al. (2016) (see Supporting Information). We characterized the δ^13^C and δ^15^N values of 12 amino acids: alanine (Ala), glycine (Gly), threonine (Thr), serine (Ser), valine (Val), leucine (Leu), isoleucine (Ile), proline (Pro), aspartic acid (Asp), glutamic acid (Glu), phenylalanine (Phe) and lysine (Lys). Acid hydrolysis converts glutamine (Gln) and asparagine (Asn) into Glu and Asp, respectively, resulting in the measurement of combined Gln + Glu (hereafter referred to as Glx) and Asn + Asp (hereafter referred to as Asx). For δ^15^N analyses, we defined Glx, Asx, Ala, Leu, Ile, Pro and Val as trophic amino acids, and Phe and Lys as source amino acids. Gly, Ser, and Thr were kept as separate groups due to a lack of consensus on their classification (McMahon & McCarthy, 2016). For δ^13^C analyses, we defined Thr, Val, Leu, Ile, Phe and Lys as essential amino acids and all other amino acids as non‐essential amino acids (McMahon et al., 2010). Mean reproducibility of the laboratory mixed amino acid standard was ±0.79‰ for δ^13^C and ±0.69‰ for δ^15^N, calculated as the SD of means across all sample runs and averaged across all individual amino acids.

We first used linear discriminant analysis to confirm differentiation in δ^13^C_EAA_ fingerprints between carbon sources, then used δ^13^C_EAA_ fingerprinting and Bayesian stable isotope mixing models to quantify the proportional contributions of carbon sources to carnivorous fish food webs and assess variation in relation to human disturbance level. We built species‐specific Bayesian stable isotope mixing models using MixSIAR (Stock et al., 2018). We used the mean and standard deviation of δ^13^C_EAA_ values of six essential amino acids (Ile, Leu, Lys, Phe, Thr, Val) from the carbon source proxies as the source groups. The trophic discrimination factor (TDF) was set to 0.1 ± 0.1‰ (McMahon et al., 2010). We used δ^13^C_EAA_ values from individual carnivorous fish for the consumer data. We ran the models using an uninformative prior, multiplicative error (process × residual error), and the ‘very long’ Markov chain Monte Carlo settings. We assessed model convergence using Gelman‐Rubin and Geweke diagnostics. We used resulting posterior probability distributions to estimate the proportional contributions of carbon sources to carnivorous fish food webs. It is important to acknowledge that for secondary consumers, this approach quantifies the contribution of carbon sourced from each baseline end member to the food web supporting each consumer and does not suggest that those consumers were feeding directly on those basal end members.

We calculated trophic positions (TP_CSIA_) for individual fish using the TP_CSIA_ equation and the simple mean δ^15^N values of seven trophic (Ala, Val, Leu, Ile, Pro, Asx, Glx) and two source (Phe, Lys) amino acids (Chikaraishi et al., 2009; see Supporting Information). *β* values were derived from Ramirez et al. (2021) (Avg_TrophicAA_–Avg_SourceAA_ = 3.0 ± 2.4‰), whereas trophic discrimination factor values were derived from Bradley et al. (2015) (Avg_TrophicAA_–Avg_SourceAA_ = 5.5 ± 0.5‰). Errors were propagated using the *propagate* package in R (Spiess, 2018).

We used general linear models (GLMs) to test whether TP_CSIA_ varied as a function of human disturbance level. Species‐specific models included TP_CSIA_ as the response variable and human disturbance level (continuous) as the explanatory variable. Preliminary data exploration revealed a positive relationship between TP_CSIA_ and standard length for most fish species (Figure S1). As a result, prior to statistical analyses, we normalized individual fish TP_CSIA_ estimates to the mean standard length for each species (Figure S1). Shapiro–Wilk's and Levene's tests confirmed the species‐specific data were normally distributed and homoscedastic. All statistical analyses were performed in R 4.1.1 (R Core Team, 2021) interfaced with RStudio 2022.02.3+492 (RStudio Team, 2022).

## 3 RESULTS

### 3.1 δ^13^C_EAA_ fingerprinting

**FIGURE 2:**
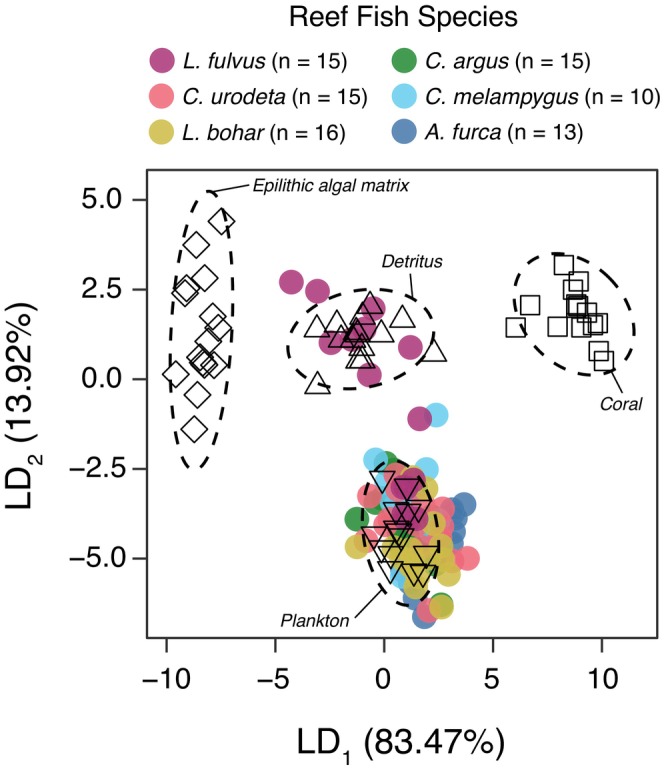
Linear discriminant analysis of δ^13^C values of six essential AAs (Thr, Val, Leu, Ile, Phe, Lys) for six carnivorous reef fish species and four carbon source end members. Data for all human disturbance levels are combined. Dashed lines represent 95% confidence ellipses around each source group.

**FIGURE 3:**
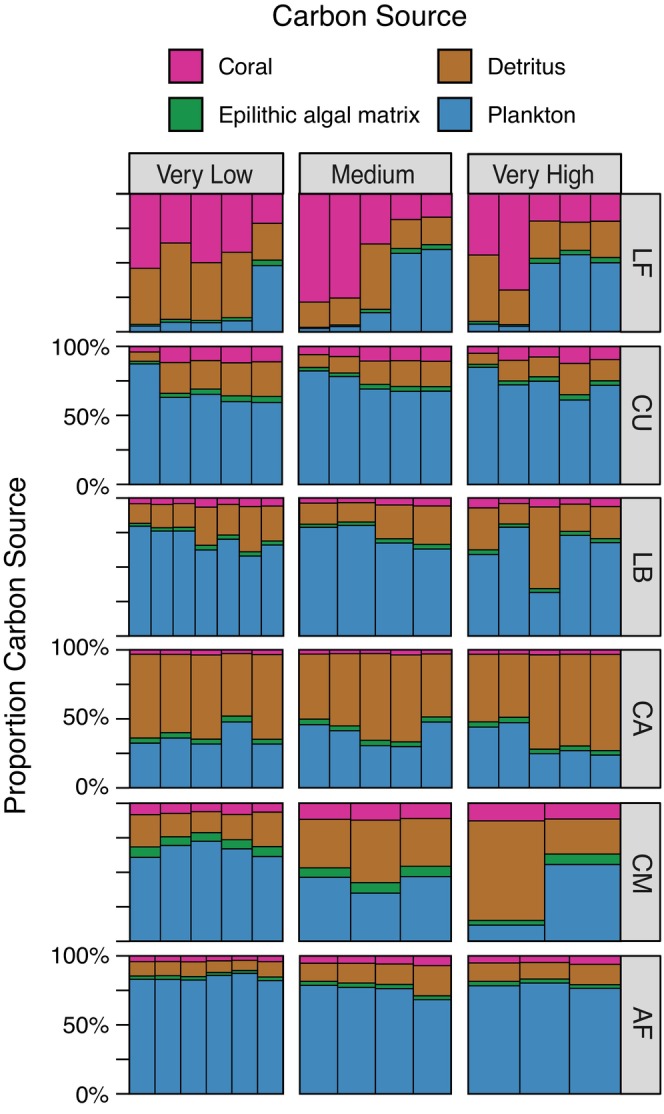
Proportional contributions of four basal production sources to individual carnivorous reef fish (bars within panels) on coral reefs with different human disturbance levels. Source contributions were estimated using Bayesian stable isotope mixing models. Coloured bars represent mean values for individual fish, ordered by increasing body size within species‐specific panels. Generalist carnivores: LF = *L. fulvus*, CU = *C. urodeta*. Piscivores: LB = *L. bohar*, CA = *C. argus*, CM = *C. melampygus*, AF = *A. furca*.

The linear discriminant analysis had 100% reclassification success rates for all carbon sources, confirming high differentiation in δ^13^C_EAA_ fingerprints (Figure 2; see Supporting Information). The proportional contribution of the four carbon sources—benthic coral, microbially recycled detritus, epilithic algal matrix, water column phytoplankton—to carnivorous fishes varied among species but was generally consistent within a species among very low, medium, and very high disturbance sites (Figure 3; Tables S2 and S3). The only species that exhibited possible carbon source variation with human disturbance level was the piscivore *C. melampygus* that exhibited increasing contribution of detrital carbon with increasing disturbance level (Figure 1; Table S2), although inferences are limited for this species due to small sample sizes relative to the other sampled species.

Five of the six species received >80% of their carbon from phytoplankton and detritus‐based energy channels (Figure 3; Figure S2). The mixing model results suggested that planktonic sources provided a disproportionate amount of carbon to individual *A. furca* (76%–100%), *L. bohar* (32%–80%), and *C. urodeta* (59%–87%), followed by detrital sources (7%–59%) (Table S2). In contrast, for the piscivore *C. argus*, the dominant carbon source was detritus (45%–70%), followed by plankton (30%–48%). Planktonic (12%–72%) and detrital (15%–72%) carbon contributions were similarly high across sampled *C. melampygus* but were more variable across the disturbance gradient. For these five species, coral and epilithic algal matrix supplied 3%–13% and 3%–8% of assimilated carbon, respectively. Relative to the other species, the generalist carnivore *L. fulvus* exhibited much greater variation in carbon source contributions among individuals and between sampling sites (coral: 17%–69%; detritus: 18%–55%; plankton: 2%–60%) (Figures S2 and S3), though not disturbance levels, which appear to be due to an ontogenetic shift in carbon source reliance from coral and detritus to plankton with increasing size (Table S2).

### 3.2 Trophic ecology

**FIGURE 4:**
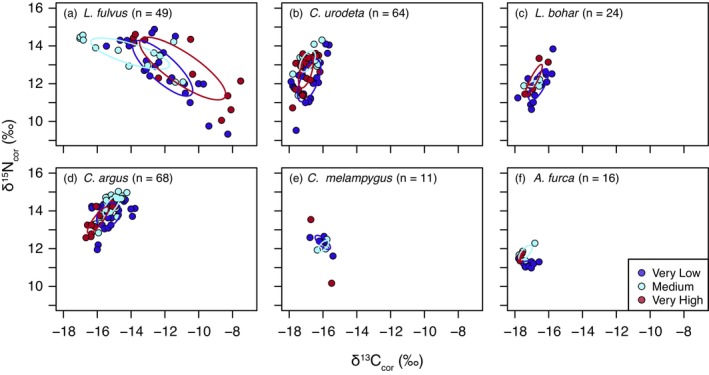
Baseline‐corrected bulk tissue stable carbon (δ^13^C) and nitrogen (δ^15^N) isotope values for carnivorous reef fishes by human disturbance level. Solid lines represent sample size‐corrected Standard Ellipse Areas (SEAc). SEAs are only reported for disturbance levels with *N* ≥ 3. See Table S1 for disturbance level‐specific sample sizes.

Carnivorous fish isotopic niche widths and positions—widely used proxies for ecological niche (Newsome et al., 2007)—did not vary systematically across the human disturbance gradient. Within a species, SEAs were generally consistent in size (mean SEAc = 0.04 to 2.00‰^2^ for all but *L. fulvus*; Figure 4; Figure S4; Tables S3 and S4) and tended to exhibit high overlap in bivariate isotope space among human disturbance levels (mean overlap 54%–75% for all but *A. furca*; Table S5). For *C. urodeta*, *L. bohar* and *C. argus*, isotopic niche size tended to be larger in very low relative to medium disturbance sites, but within‐species isotopic niche overlap among disturbance levels remained high (38%–94%; Table S5). The generalist carnivore *L. fulvus* exhibited bulk tissue stable isotope patterns that were distinct from the other species, having relatively large isotopic niches across sites (mean SEAc = 4.38 to 8.51‰^2^; Figure S2; Table S3) and distinct changes in δ^13^C and δ^15^N values with increasing size (Figure S5). When combined with the δ^13^C_EAA_ fingerprinting data, these results reinforce that this variation for *L. fulvus* reflects ontogenetic changes in resource use independent of the influence of human disturbance.

**FIGURE 5:**
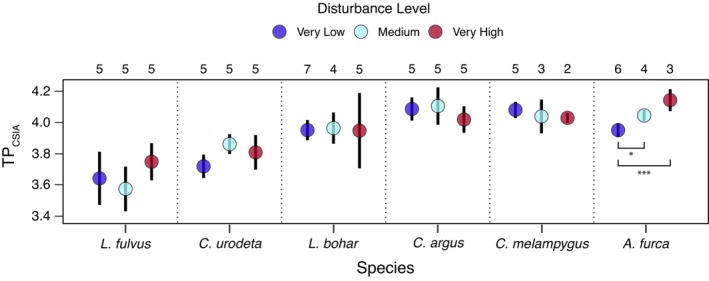
Mean ± 95% confidence intervals for size‐corrected trophic position (TP_CSIA_) estimates by carnivorous reef fish species across the human disturbance gradient. Sample sizes are presented at the top of each panel. See Figure S6 for site‐specific data.

Trophic positions of carnivorous fishes also varied little as a function of human disturbance (Figure 5; Table 2). TP_CSIA_—standardized to mean standard length within species—ranged between 3.3 and 4.3 across all sampled fishes, with variation among individuals spanning only 0.23 to 0.72 trophic levels (mean = 0.44) for each species that was generally consistent across disturbance sites (Figure 5; Table 2). Variation in mean TP_CSIA_ between disturbance levels was also low for each species, with the mean difference in mean TP_CSIA_ between all combinations of disturbance sites being 0.07 trophic levels (range = 0 to 0.2). Unlike the other species, the piscivore *A. furca* exhibited a statistically significant positive relationship between TP_CSIA_ and human disturbance level (*R* ^2^ = 0.65, *F*_1,11_ = 23.2, *p* < 0.001), with TP_CSIA_ spanning 0.72 trophic levels among individuals and increasing by 0.2 trophic levels between very low and very high disturbance sites. Additionally, trophic position estimates for the generalist carnivore *L. fulvus* were more variable than those of the other species, though these did not change consistently with disturbance level, mirroring the high variation in bulk tissue δ^13^C and δ^15^N values observed for this species (Figures S5 and S6).

**TABLE 2:** Statistical results for general linear models of the relationships between body‐size‐corrected trophic position (corrected TP_CSIA_) and human disturbance level (DL, continuous variable).

Species	*n*	AIC	Adj. *R* ^2^	*F*	df	Variable	Est.	SE	*t*‐value	*p*‐value
*Lutjanus fulvus*	15	−6.6	0.01	1.14	1, 13	DL	0.064	0.060	1.07	0.305
*Cephalopholis urodeta*	15	−19.4	−0.04	0.41	1, 13	DL	0.023	0.036	0.64	0.534
*Lutjanus bohar*	16	−8.3	−0.07	0.00	1, 14	DL	−0.004	0.062	−0.06	0.950
*Cephalopholis argus*	15	−21.1	0.04	1.62	1, 13	DL	−0.04	0.028	−1.27	0.226
*Caranx melampygus*	10	−22.1	−0.04	0.68	1, 8	DL	−0.021	0.025	−0.83	0.433
*Aphareus furca*	13	−34.7	0.65	23.2	1, 11	DL	0.121	0.025	4.82	<0.001

## 4 DISCUSSION

Human disturbance is expected to alter species interactions, thereby modifying key food web attributes, including energy pathways, food chain length, and ecological niches. Hyperdiverse tropical systems are heavily impacted by anthropogenic stressors almost universally, and yet the extent to which human activities alter the flow of energy through them remains poorly understood. By leveraging novel molecular isotope techniques and an ecosystem‐scale natural experiment, we show that multiple facets of food web architecture, including carbon pathways, trophic dynamics, and aspects of ecological niche can remain stable in the face of human disturbance. Specifically, we show that the common and socio‐culturally important generalist carnivorous reef fish species we sampled consistently foraged in highly siloed energy channels, maintaining consistency in baseline carbon sources, isotopic niche width and position, and trophic position despite substantial disturbance‐mediated shifts in benthic community composition (Baum et al., 2023), foraging habitat structural complexity (Magel et al., 2019), and fish community assemblages (Magel et al., 2020) (Figure 1). While human disturbance certainly impacts a wide range of ecological processes, our results suggest that important food web attributes that connect basal resources to carnivorous reef fishes can be conserved for some species positioned within plankton and detrital energy channels following anthropogenically driven loss of reef‐building foundation species.

Following disturbance, species loss and turnover are expected to reroute energy through existing food web connections (Bartley et al., 2019). For example, warming, drought, and terrestrial nutrient inputs are known to reconfigure energy fluxes within temperate and tropical aquatic systems (Allgeier et al., 2020; Hempson et al., 2017; Ledger et al., 2013, p. 201; O'Gorman et al., 2019). In line with this body of work, we hypothesized that human disturbance‐mediated changes in benthic community composition and complexity in Kiritimati would reconfigure energy flow pathways, simplifying the system from one supported by diverse baseline energy sources to one supported by fewer taxa (e.g. epilithic algal matrix, plankton). However, we observed stability in the carbon pathways connecting basal resources to five of the six sampled upper trophic level carnivorous fish species (piscivores: *A. furca*, *C. argus*, *L. bohar*; generalist carnivores: *C. urodeta*, *L. fulvus*), which suggests minimal rewiring of carbon pathways supporting these species. Our results mirror recent research showing that land use change does not universally alter carbon source contributions within tropical freshwater systems (Wilkinson et al., 2021), but contrast with the handful of studies that have found that habitat degradation alters carbon flow within coral reef food webs. For example, in the Caribbean and southern Great Barrier Reef, Australia, coral loss has led to shifts in basal carbon sourcing in carnivores between benthic and pelagic resources (Hempson et al., 2017; Morillo‐Velarde et al., 2018). In the Red Sea, plankton were the primary carbon source for meso‐predator Ehrenberg's snapper (*Lutjanus ehrenbergii*) on oceanic reefs (72% contribution) with high coral cover, but macroalgae (71% contribution) were the primary carbon source in coastal shelf reefs where macroalgae dominated the benthos (McMahon et al., 2016). Herein, the general consistency of within‐species carbon sourcing across sites combined with the lack of significant algal and coral contributions to most sampled fish suggest energetic pathways linked to carnivorous reef fish may be conserved for some taxa in Kiritimati, an oceanic atoll subject to island‐wake upwelling, across gradients of chronic local human disturbance.

New water column planktonic production and microbially reprocessed detritus appear to be key production sources mediating the stability of carnivorous reef fish energy channels on Kiritimati. For five of the six sampled reef fishes, phytoplankton and detrital channels combined to provide >80% of their carbon, with four reef fish species exhibiting a consistent and disproportionate (>50%) reliance on either planktonic (*A. furca*, *L. bohar*, *C. urodeta*) or detrital (*C. argus*) carbon. *C. melampygus* exhibited a shift in carbon acquisition from planktonic to detrital sources along our disturbance gradient. *L. fulvus* was the only species to show the significant integration of multiple energy channels across sites, as might be expected of an upper trophic level generalist (Rooney & McCann, 2012). This apparent lack of energy channel coupling across the sampled species contrasts with findings in other marine, freshwater, and terrestrial systems, where multichannel feeding (i.e. consumption across both autotroph‐based ‘green’ and detritus‐based ‘brown’ energy pathways) is common and considered a stabilizing feature of food webs (Rooney et al., 2006; Wolkovich et al., 2014). One potential explanation for this pattern is the absence of top predators such as elasmobranchs in our sampling, a common limitation in food web studies (Pringle & Hutchinson, 2020), which may obscure energy channel coupling occurring at higher trophic levels (e.g. seen in giant moray eels, *Gymnothorax javanicus*, in the Red Sea, McMahon et al., 2016). Species‐specific foraging behaviour also provides some insight into our observed patterns. *A. furca'*s strong reliance on plankton aligns with its high mobility and use of pelagic habitats (Filous et al., 2017), whereas similar planktonic reliance in reef‐associated species like *C. urodeta*, *L. bohar* and *C. argus* may suggest opportunistic feeding given that they typically have access to benthic resources (Myers, 1999; Ticzon et al., 2012). The dominance of planktonic channel feeding among these species may reflect the persistence of oceanic upwelling that sustains the planktonic energy base, especially given that planktivores comprised ~80% of total fish abundance across the atoll (Figure 1) (Magel et al., 2020). Detrital reliance in *C. melampygus* likely reflects its predation on small, diurnally active fishes within protected reef zones (Meyer et al., 2001). Interestingly, our δ^13^C_EAA_ fingerprinting analysis suggests that, with the exception of *L*. *fulvus*, the fish species studied herein ultimately derive their carbon from phytoplankton, either directly or indirectly through the detrital energy channel. This result supports an emerging body of work demonstrating significant detrital contributions to coral reef meso‐predators, including through water column zooplanktivores, constituting a probable mechanism of detrital energy transfer to meso‐predators (McMahon et al., 2016; Tietbohl, 2016).

The importance of planktonic carbon sources in our study aligns with a growing body of evidence that planktonic production is a key source of energy supporting carnivorous reef fishes globally (Zgliczynski et al., 2019), which has been observed in oceanic contexts like Kiritimati Atoll as well as the Red Sea. Early studies hypothesized that aggregations of reef‐associated planktivores form a ‘wall of mouths’ on coral reefs that sequester significant amounts of oceanic carbon into fish biomass (Emery, 1973; Hamner et al., 1988). Multiple bulk tissue stable isotope studies have suggested this for years (Frisch et al., 2014; McCauley et al., 2012; Skinner et al., 2019), though the interpretation of bulk tissue SIA data in a food web context in complex reefs is quite challenging (McMahon et al., 2016; Skinner et al., 2022). Our study, in conjunction with three other recent δ^13^C_EAA_ fingerprinting studies documenting high reliance (>60%) of upper trophic level coral reef predators on planktonic production in the Red Sea and the Maldives (McMahon et al., 2016, 2025; Skinner et al., 2021), advances long‐standing hypotheses regarding tight trophic linkages among well‐mixed oceanic production, planktivores and upper trophic level consumers (Emery, 1973; Hamner et al., 1988).

Although inferences are limited to the species sampled herein, this high reliance on planktonic production among carnivores also suggests the possible existence of trophic ‘dead ends’ in Kiritimati, whereby certain basal resources may support distinct, short food webs ending with dietary specialists that do not substantially contribute to the biomass of carnivores (e.g. corallivores, large‐bodied herbivores). For example, the absence of an epilithic algal matrix carbon signal for most fish is notable given that herbivores comprise 35.5% of fish biomass in Kiritimati and that benthic turf algae is the dominant algal source in this system (Figure 1; Table 1). This could be attributed to the fact that a few, large‐bodied herbivores from the genera *Scarus* and *Acanthurus* contribute disproportionately to herbivore biomass on Kiritimati (e.g. eight of 35 observed species ≈ 75% herbivore biomass). Notably, the average body mass for seven herbivores that account for ~50% of herbivore biomass exceeds the average body mass for *A. furca*, *L. fulvus* and *C. urodeta*. Although these species may be accessible food sources as juveniles, on average these species may be inaccessible prey for all but the largest piscivores (e.g., *L. bohar*, *C. melampygus* and some *C. argus*) (Dunic & Baum, 2017). Broader food web sampling for δ^13^C_EAA_ on Kiritimati, and on coral reefs in general, would aid in evaluating this hypothesis.

Even within food webs with robust carbon pathways, human disturbance is expected to reorganize trophic structure in response to changes in diversity and abundance (O'Gorman et al., 2019), community composition (Morais et al., 2020), predator–prey body size ratios (Jennings & Warr, 2003) and trophic niches (Letourneur et al., 2017). Such changes in trophic ecology should be particularly evident in mobile generalist predators like those examined in this study that, unlike specialist consumers, have the capacity to alter their distributions and/or foraging patterns to adapt to disturbance‐driven changes in resource availability (Bartley et al., 2019), promoting their persistence (Lu et al., 2016). For example, warming causes the disproportionate loss of consumers relative to resources in temperate streams, leading to food web simplification and reduced connectivity (Ledger et al., 2013; Lu et al., 2016; O'Gorman et al., 2019). In tropical forests, mammal behavioural plasticity expands isotopic niches in human‐modified landscapes relative to natural habitats (Magioli et al., 2019). In contrast, in tropical streams, land use change does not affect predator isotopic niche width but differentially impacts apex and meso‐predator trophic positions (Wilkinson et al., 2021), reducing maximum food chain length. On tropical coral reefs, coral loss and regime shifts can reduce meso‐predator and piscivore (*C. argus*) trophic position, isotopic niche size, and nutritional condition (Hempson et al., 2017; Hempson, Graham, MacNeil, Bodin, et al., 2018; Hempson, Graham, MacNeil, Hoey, et al., 2018). Habitat degradation elicits a variety of trophic responses in lower order marine fish trophic guilds (Letourneur et al., 2017; Wilson et al., 2006).

However, mirroring the consistency in carbon source contributions, we found that isotopic niche characteristics and trophic position were strikingly similar for the six carnivorous reef fish species in this study across the major human‐disturbance gradient. These patterns contrast with significant disturbance‐associated variation in reef fish community structure in Kiritimati (Magel et al., 2020), with herbivores, invertivores, planktivores, and piscivores displaying high variation in site‐level biomass across the atoll (Figure 1). This consistency in trophic metrics across carnivores challenges classic paradigms of dramatic disturbance‐driven changes in community composition driving bottom‐up changes in carnivore trophic dynamics. Our results, instead, add new evidence to an emerging view that some food web attributes appear robust enough to withstand large‐scale coral losses and biodiversity shifts. For example, Morillo‐Velarde et al. (2018) found that the isotopic niches and trophic positions of a range of consumer groups—herbivore, invertivore, carnivore—were similar on adjacent coral reefs with contrasting levels of degradation in the Mexican Caribbean. On coral atolls in the Lakshadweep Archipelago, Indian Ocean, the piscivore *C. argus* maintained its trophic niche following bleaching‐mediated declines in reef structural complexity by switching from ambush to roving feeding modes and expanding its foraging territory size (Karkarey et al., 2017). These food web attributes could be maintained through behavioural mechanisms (e.g. expanded territories or prey switching within isotopically well‐mixed feeding guilds) or preservation of small predator–prey body size ratios across the disturbance gradient (Jennings & Warr, 2003).

Collectively, our work, which simultaneously tracked carbon sourcing and trophic ecology, suggests that robust planktonic and detrital energy channels and not dietary plasticity may be a key mechanism buffering the focal carnivorous reef fishes from specific deleterious effects of chronic local disturbance on Kiritimati atoll. Most sampled fishes appear to draw from a narrow range of resources linked to foraging trophic level and/or microhabitat regardless of pronounced variations in resource availability across the disturbance gradient. As a result, these carnivorous reef fishes may be considered nominally generalist, utilizing a narrower range of resources than conventionally believed. These findings do not preclude carnivores, both those sampled herein and others, from preying upon a diverse array of herbivores that target different basal resources such as macroalgae (McMahon et al., 2016, 2025) or offshore and nearshore planktivores (Skinner et al., 2021), which were not evaluated in our study. Although robust planktonic and detrital energy channels have clear benefits to maintaining biodiversity, high reliance on a single energy channel may ultimately make these nominally generalist species more susceptible to anthropogenic disturbance than conventionally considered for a dietary generalist (McMahon et al., 2019). Of particular concern in Kiritimati is if pelagic energy channels destabilize, for example through ocean warming‐induced reductions in production (Lo‐Yat et al., 2011), mismatched shifts in phytoplankton bloom phenology (Gittings et al., 2018), and shifts in plankton community composition towards small‐celled cyanobacterial production (Karati et al., 2017).

## 5 CONCLUSIONS

Empirical evidence and theory suggest that the loss of diverse energy channels or mobile consumers should destabilize diverse food webs (Rooney et al., 2006). However, we demonstrate that dramatic human disturbance‐mediated changes in benthic community composition, including the loss of reef‐building corals, foraging habitat structural complexity, and fish community assemblages, do not universally alter the broad energy fluxes and trophic relationships connecting basal resources to upper trophic level generalist consumers. Our findings suggest that, in contrast to theoretical predictions, maintenance of a few dominant production sources can stabilize certain food web attributes in hyperdiverse systems. On coral reefs, specifically, robust planktonic and detrital energy channels may provide a mechanism that promotes stability to other food web attributes, buffering some species from certain deleterious effects of human disturbance. Although our inferences may be constrained by our sampling design, the six sampled carnivorous reef fish species comprise almost half of the atoll's carnivorous fish biomass and thus inferences likely apply to this system's food web. As a result, mitigation strategies that restore habitats, lessen human pressures (e.g. improve water quality, reduce fishing pressure), and sustain pelagic energy channels may allow for the maintenance and recovery of key ecosystem processes on degraded reefs. Such actions are particularly important in marine resource‐dependent Small Island Developing States, such as Kiribati (Watson et al., 2016), whose high reef dependence for sustenance and livelihoods makes them disproportionately vulnerable to climate change (IPCC, 2022).

## AUTHOR CONTRIBUTIONS

Julia Baum coordinated and funded the fieldwork. Julia Baum and Kelton McMahon funded the laboratory research. Matthew Ramirez and Kelton McMahon designed the methodology and analysed the data. Matthew Ramirez performed the laboratory work and statistical analyses and led manuscript writing. All authors conceived the study, provided input on data interpretation, contributed critically to manuscript drafts, and gave final approval for publication.

## CONFLICT OF INTEREST STATEMENT

The authors declare that they have no conflict of interest.

## STATEMENT ON INCLUSION

Our study does not include scientists based on Kiritimati because of the limited capacity for the few government scientists to participate in non‐fisheries‐related research. Over a decade of research in the Republic of Kiribati, we have instead worked to develop relationships with managers in the Ministry of Fisheries and Ministry of Environment, Lands and Agriculture Development, including offering underwater science training opportunities, providing our data, developing and sharing annual research reports, and in‐person meetings while on island. We recognize that more could always be done to engage local scientists and to embed our research with Kiribati's research priorities, and we are working to strengthen our relationships with the aim of one day co‐developing coral reef research.

## Supporting information


**Figure S1.** Body size‐trophic position relationships for carnivorous reef fishes.
**Figure S2.** Proportional contributions of four basal production sources to individual carnivorous reef fish (clustered boxplots within panels) on coral reefs with different human disturbance levels.
**Figure S3.** Sampling site‐specific mean ± 95 % confidence intervals for contributions of four carbon source end‐members (coral, detritus, epilithic algal matrix, plankton) to two generalist carnivore (*L. fulvus*, *C. urodeta*) and four piscivore (*L. bohar*, *C. argus*, *C. melampygus*, *A. furca*) reef fish on Kiritimati Atoll.
**Figure S4.** Isotopic niche sizes for carnivorous reef fishes.
**Figure S5.** Body size‐stable isotope relationships for carnivorous reef fishes.
**Figure S6.** Sampling site‐specific mean ± 95% confidence intervals for size‐corrected trophic position (TP_CSIA_) estimates by carnivorous reef fish species across the human disturbance gradient.
**Figure S7.** Source amino acid stable nitrogen isotope (δ^15^N) values by species, human disturbance level (upper panels), and sampling site (lower panels).
**Table S1.** Characteristics of fish sampled via bulk stable isotope analysis (SIA) and compound‐specific stable isotope analysis of amino acids (CSIA‐AA).
**Table S2.** Posterior probabilities of classification of carnivorous fish via simple LDA, bootstrapped LDA (LDA_boot_), and Bayesian Stable Isotope Mixing Model (SIMM).
**Table S3.** Output from SIBER models comparing baseline corrected bulk tissue stable carbon (δ^13^C) and nitrogen (δ^15^N) isotope values for carnivorous reef fish by human disturbance level.
**Table S4.** Comparisons of isotopic niche size among human disturbance levels.
**Table S5.** Comparisons of isotopic niche overlap among human disturbance levels.
**Table S6.** Linear discriminant coefficients for the LDA model using δ^13^C values of six essential AAs (Ile, Leu, Ly, Phe, Thr, Val) for reef fish and carbon source end‐members.
**Table S7.** Correlations between pairs of carbon sources from species‐specific Bayesian Stable Isotope Mixing Models.

## Data Availability

All data and R scripts needed to evaluate the conclusions in the paper and reproduce the analyses are presented in the paper, Supporting Information S1, Github (https://github.com/matthewdramirez/Ramirez_etal_2025_JAE), or Zenodo (Ramirez et al., 2025; https://doi.org/10.5281/zenodo.17068835).
