# Supplementary material for: Carbon pathways and trophic attributes are conserved in carnivorous reef fishes across a major human disturbance gradient
Source: J Anim Ecol. 2025 Oct 12;95(1):39–53. doi: 10.1111/1365-2656.70151 (PMC12775554; doi:10.1111/1365-2656.70151)
Supplement: Supplementary file 1 — Figure S1. Body size‐trophic position relationships for carnivorous reef fishes. Figure S2. Proportional contributions of four basal production sources to individual carnivorous reef fish (clustered boxplots within panels) on coral reefs with different human disturbance levels. Figure S3. Sampling site‐specific mean ± 95 % confidence intervals for contributions of four carbon source end‐members (coral, detritus, epilithic algal matrix, plankton) to two generalist carnivore (L. fulvus, C. urodeta) and four piscivore (L. bohar, C. argus, C. melampygus, A. furca) reef fish on Kiritimati Atoll. Figure S4. Isotopic niche sizes for carnivorous reef fishes. Figure S5. Body size‐stable isotope relationships for carnivorous reef fishes. Figure S6. Sampling site‐specific mean ± 95% confidence intervals for size‐corrected trophic position (TPCSIA) estimates by carnivorous reef fish species across the human disturbance gradient. Figure S7. Source amino acid stable nitrogen isotope (δ15N) values by species, human disturbance level (upper panels), and sampling site (lower panels). Table S1. Characteristics of fish sampled via bulk stable isotope analysis (SIA) and compound‐specific stable isotope analysis of amino acids (CSIA‐AA). Table S2. Posterior probabilities of classification of carnivorous fish via simple LDA, bootstrapped LDA (LDAboot), and Bayesian Stable Isotope Mixing Model (SIMM). Table S3. Output from SIBER models comparing baseline corrected bulk tissue stable carbon (δ13C) and nitrogen (δ15N) isotope values for carnivorous reef fish by human disturbance level. Table S4. Comparisons of isotopic niche size among human disturbance levels. Table S5. Comparisons of isotopic niche overlap among human disturbance levels. Table S6. Linear discriminant coefficients for the LDA model using δ13C values of six essential AAs (Ile, Leu, Ly, Phe, Thr, Val) for reef fish and carbon source end‐members. Table S7. Correlations between pairs of carbon sources from species‐specific Bay [file JANE-95-39-s001.docx]

**Supporting Information**

**Carbon pathways and trophic attributes are conserved in carnivorous reef fishes across a major human disturbance gradient**

**Methods**

***Carbon source proxies: specialist consumers***

We chose four specialized primary consumers based on dietary information from the published literature and their prevalence on Kiritimati Atoll to serve as proxies for four distinct basal production carbon sources (n = 5 per disturbance level per species; 60 total fish): coral (obligate corallivore: ornate butterflyfish, *Chaetodon ornatissimus*), detritus (detritivore: striped-fin surgeonfish, *Ctenochaetus marginatus*), epilithic algal matrix (herbivore: whitecheek surgeonfish, *Acanthurus nigricans*), and phytoplankton (planktivore: olive anthias, *Pseudanthias olivaceus*). *C. ornatissimus* is an obligate corallivore that feeds strictly on coral polyps or mucus (Harmelin-Vivien & Bouchon-Navaro, 1983), so is expected to serve as a suitable coral carbon source proxy similar to the closely related *C. trifascialis* (McMahon et al., 2016). *C. marginatus* is primarily a detritus and sediment feeder (Randall, 2005). Multiple CSIA studies have found closely related *C. striatus* to be a suitable detrital carbon source proxy (McMahon et al., 2016; Skinner et al., 2021). *A. nigricans* is an herbivorous grazer that feeds principally on turfing, filamentous, and small thallate algae (Choat et al., 2002), which are present with the epilithic algal matrix that also includes non-living organic components (detritus), microbial components, and inorganic material (sediment) (Wilson & Bellwood, 1997). Herbivore feeding trials conducted on Kiritimati indicated this species is feeding solely on benthic turf algae (Kindinger et al., 2024). Benthic turf algae are the primary algal source and second most common organic benthic substrate on Kiritimati, comprising 16.4 ± 6.4 % of the benthos across sampling sites (Baum et al., 2023) (Tables 1 and S13). Fleshy macroalgae comprise only 3.9 ± 2.9 % of the benthos across sampling sites. *Acanthurus* sp. consistently provide the greatest proportion of algal consumption across Pacific coral reefs (Kindinger et al., 2024), making them an appropriate proxy for the epilithic algal matrix. Closely related *C. leucostrenon* has been previously used as an algal carbon source proxy (Skinner et al., 2021). *P. olivaceus* is considered a diurnal zooplanktivore (Randall, 2005). Although these fish proxies do not integrate all possible species within a production class, they are assumed to reflect the dominant broad production sources on Kiritimati Atoll and thus provide a broad resolution picture of carbon flow in this system.

Moreover, the proxy approach has some very critical benefits that make it the preferred approach in CSIA-based food web studies of biodiverse systems [see examples in Fox et al. (2019); McMahon et al. (2016); Skinner et al. (2021); Tietbohl (2016)]. First, carbon source proxies with well characterized and constrained diets provide an integrated signal of primary production isotope dynamics that better align with the comparable turnover rates of our target meso-predator fishes. As a result, this approach avoids issues of trying to match short time scale, highly variable primary producer isotope dynamics with the multi-month integration times of our target meso-predator fishes (Whiteman et al., 2019). Second, isotope fingerprints recorded in fish tissue reflect the actual assimilated carbon source signal, which is what is in turn passed on to upper trophic level consumers. Analyzing the primary producers themselves will provide a mix of both assimilated and non-assimilated isotope signals. Lastly, there are significant and reproducible differences in multivariate amino acid isotope signals among major production sources (phytoplankton, macroalgae, coral, and detritus) and these among group differences are far larger than within group differences both within coral reefs (Fox et al., 2019; McMahon et al., 2016; Skinner et al., 2021; Tietbohl, 2016) and other systems (Besser et al., 2022; Elliott Smith et al., 2022; Manlick & Newsome, 2022; Rowe et al., 2019). As such, while a proxy species may miss a specific producer species, it is unlikely that their amino acid carbon isotope fingerprints will be misclassified as a different major production source.

***Carnivorous fish habitat associations and home ranges***

We sampled six of the most abundant and socio-culturally important carnivorous reef fish species on Kiritimati (Walsh, 2011), including four piscivores (small toothed jobfish, *Aphareus furca*; bluefin trevally, *Caranx melampygus*; peacock hind, *Cephalopholis argus*; two-spot red snapper, *Lutjanus bohar*) and two generalist carnivores (blacktail snapper, *Lutjanus fulvus;* darkfin hind, *Cephalopholis urodeta*). All species have diets dominated by fish that are supplemented with benthic or pelagic invertebrates. Although all six species are associated with coral reefs, they have different habitat associations within reef systems that may influence their trophic ecology and carbon source patterns.

*A. furca* and *C. melampygus* are generally considered the most transient of the species sampled and thus most likely to utilize planktonic energy channels due to their relatively high use of both benthic and pelagic habitats (Mundy, 2005). Although *C. melampygus* makes high use of pelagic habitats, it is also strongly reef-associated for foraging and primarily feeds on small diurnally active fish found within protected reef zones (Meyer et al., 2001; Sudekum et al., 1991). Multiple studies have observed *C. melampygus* to have high site fidelity where they make regular diurnal movements between shallow-day and deep-night habitats. Tracking studies suggest *C. melampygus* generally have home ranges < 1 km^2^ (Filous et al., 2017; Holland et al., 1996; Meyer & Honebrink, 2005), although individuals are occasionally tracked traveling > 10 km between release and recapture sites (Holland et al., 1996; Meyer & Honebrink, 2005). *A. furca* movement is poorly studied. Tagging of the closely related green jobfish (*Aprion virescens*) significantly lower site fidelity than *C. melampygus*, with 50% of recaptures occurring 2.5–30 km from release sites (Filous et al., 2017).

*C. argus* and *C. urodeta* are strongly associated with a variety of inner-reef microhabitats, including coral rubble, pavement, and branching microhabitats (Donaldson, 2002; Nanami, 2021; Shpigel & Fishelson, 1989; Ticzon et al., 2012). *L. bohar* tends to occupy outer reef habitats as well as sheltered lagoons, whereas *L. fulvus* occupies sheltered inshore reefs and lagoons (Myers, 1999). *C. argus* has a documented average home range size of 1.3 km^2^, with a maximum territory size of 2 km^2^ (Shpigel & Fishelson, 1991). Home ranges for *C. urodeta, L. bohar*, and *L. fulvus* have yet to be described but studies in other *Cephalopholis* and *Lutjanus* sp. suggest hind and snapper generally have high site fidelity and narrow home ranges (0.1 to 4.0 km^2^) that vary in size as a function of fish size and mating system (Lindholm et al., 2005; Liu & Sadovy, 2005; Luo et al., 2009; Popple & Hunte, 2005; Shpigel & Fishelson, 1991; Topping & Szedlmayer, 2011).

***Isotopic niche analyses***

Prior to isotopic niche analyses, bulk tissue δ^13^C and δ^15^N carnivore data were baseline-corrected using essential amino acid (δ^13^C; Thr, Val, Leu, Ile, Phe, Lys) and source amino acid (δ^15^N; Phe, Lys) stable isotope data generated in this study. Specifically, correction factors applied to the bulk tissue stable isotope data were the offset values required to normalize site-specific (*j*) amino acid isotope data to the mean essential amino acid or source amino acid value for each species (*i*) across sampling sites.

$$\delta^{13}C_{cor [i,j]}= \delta^{13}C_{bulk [i,j]}+[average\left( \delta^{13}C_{EAA [i]} \right)-average\left( \delta^{13}C_{EAA [j]} \right)]$$

$$\delta^{15}N_{cor [i,j]}= \delta^{15}N_{bulk [i,j]}+[average\left( \delta^{15}N_{SourceAA [i]} \right)-average\left( \delta^{15}N_{SourceAA [j]} \right)]$$

We chose this baseline correction approach because (1) essential amino acids (δ^13^C) and source amino acids (δ^15^N) minimally fractionate with trophic transfer, thereby providing a faithful record of the baseline carbon and nitrogen signal; (2) the isotope records contained within carnivorous reef fish tissue reflect the actual assimilated carbon and nitrogen source signal for a given species at a given site; and (3) primary consumers (e.g., carbon source proxies) were sampled at many fewer sites relative to the carnivorous reef fish, impeding our ability to create correction factors using primary consumer data. Importantly, even if more broadly sampled, the use of primary producer or primary consumer data to develop a correction factor would be inappropriate within the context of this study because we clearly show there is high variation in carbon sourcing between species. Site-specific patterns of deviation from the mean also varied among primary consumers (both bulk tissue and amino acid-specific). As a result, identifying a single taxon to use for the correction would have been highly subjective. In a small number of cases (*C. argus* sites 14, 30; *C. urodeta* site 34; *L. fulvus* site 14), no site-specific amino acid isotope data were available to develop a correction factor. In these cases, we used the correction factor for an adjacent site.

For each carnivorous reef fish species, we compared isotopic niche widths and overlap among human disturbance levels using bulk δ^13^C and δ^15^N data in combination with the R packages *SIBER* (Jackson et al., 2011) and *nicheROVER* (Lysy et al., 2021). An organism's isotopic niche reflects the isotopic variation encompassed in their resource use (e.g., diet, habitat) and is thus often used as a proxy for ecological niche width (Newsome et al., 2007). We defined δ^13^C and δ^15^N as our niche axes and used standard ellipse area (SEA) as our metric of isotopic niche width, calculated for each species–disturbance level combination using both frequentist (SEAc, sample size corrected) and Bayesian approaches (SEA-B) using *SIBER*. In the Bayesian approach, the isotopic niche region was treated as a probability density function with posterior estimates calculated by iteratively fitting multivariate normal distributions to each species–disturbance level dataset via Markov chain Monte Carlo simulation. This yielded probability distributions of isotopic niche width that reflect SEA uncertainty. We ran two chains of 10,000 iterations, burning the first 1,000 draws and thinning every 10 draws.

***Compound-specific stable isotope analysis of individual amino acids (CSIA-AA)***

For a subset of the fish sampled for bulk stable isotope analysis (n = 2–7 per species per disturbance level), we analyzed muscle samples via CSIA-AA following protocols modified from McMahon et al. (2016). Muscle samples (~5–7 mg) were acid hydrolyzed in 0.5 ml of 6N HCl at 110°C for 20 hr to isolate the total free amino acids. Samples were derivatized by esterification with acidified iso-propanol followed by acylation with trifluoroacetic acid:dichloromethane (Silfer et al., 1991). The derivatized amino acids (reconstituted in 1 μl ethyl acetate) were injected into a Thermo Scientific TRACE 1310 gas chromatograph (GC) at 240°C in splitless mode and separated on a BPX5 column (60 m x 0.32 mm inner diameter, 1.0 mm film thickness; SGE Analytical Science, Austin, Texas, USA) at the University of Rhode Island, Rhode Island, USA. The separated amino acid peaks were analyzed on a Thermo Scientific Delta V Plus IRMS interfaced to the GC through a GC IsoLink II combustion furnace (1000°C) and a liquid nitrogen trap (δ^15^N analyses only). Muscle samples were analyzed in triplicate along with a mixed amino acid standard of known isotopic composition (Sigma-Aldrich Co., St. Louis, MO, USA) and a laboratory working standard (fish protein matrix). Standardization of runs was achieved using intermittent pulses of an N_2_ or CO_2_ reference gas calibrated to International Reference Standards (VPDB and N_2_). The measured isotope values of derivatized amino acids were corrected for fractionation during cumulative derivatization reactions (both C and N) and added derivative C (δ^13^C analyses only) relative to the mixed amino acid standard, a nor-Leucine internal standard of known isotopic composition added to every sample, and a working lab standard following the method of Yarnes & Herszage (2017). Mean reproducibility of the laboratory mixed amino acid standard was ± 0.79 ‰ for δ^13^C and ± 0.69 ‰ for δ^15^N, calculated as the SD of means across all sample runs and averaged across all individual amino acids.

***Comparison of carbon source contribution methods***

We used δ^13^C_EAA_ fingerprinting to identify the primary production sources supporting carnivorous fish food webs and assess variation in relation to human disturbance level. To do so, we initially used both (1) bootstrapped linear discriminant analysis (LDA), which classifies fish to carbon source groups based on the multivariate differences in δ^13^C_EAA_ values, and (2) Bayesian stable isotope mixed models, which use raw δ^13^C_EAA_ values to quantify proportional contributions of carbon sources. As Bayesian mixing models are highly influenced by undetermined mixing spaces caused by unquantified sources, LDA provides a complementary and less rigid framework to quantify source contributions that is less susceptible to underdetermined sources (Fox et al., 2019). However, a chief limitation of LDA is binary group membership relative to the more nuanced relative contribution data produced via mixing models.

We first quantified carbon source contributions to individual fish tissues using bootstrapped LDA. Within each model run of the LDA, we used the δ^13^C_EAA_ values of six essential amino acids (Ile, Leu, Lys, Phe, Thr, Val) to maximize separation among the four baseline carbon source groups, with source classification accuracy assessed using leave-one-out cross-validation to establish whether the four sources were statistically distinct. We then used this training dataset to classify individual carnivorous fish to the carbon source groups based on their δ^13^C fingerprints for the same six essential amino acids. Following Fox et al. (2019), we ran 10,000 permutations of the training dataset using random draws with replacement from the distribution of δ^13^C_EAA_ values of each source group. Each permutation of the training dataset was used to classify individual carnivorous fish to the carbon source groups. From this distribution of possible classifications, we calculated the classification percentage for each individual fish and a global classification percentage with 95% confidence interval across all sampled fish.

We then built species-specific Bayesian stable isotope mixing models using MixSIAR (Stock et al., 2018). As in the LDA, we used the mean and standard deviation of δ^13^C_EAA_ values of six essential amino acids from the carbon source proxies as the source groups in the mixing model. The Trophic Discrimination Factor (TDF) was set to 0.1 ± 0.1 ‰ given that EAAs undergo minimal trophic fractionation (McMahon et al., 2010). We used δ^13^C_EAA_ values from individual carnivorous fish for the consumer data. We ran the models using an uninformative prior, multiplicative error (process x residual error), and the “very long” Markov chain Monte Carlo settings (chain length = 1,000,000 iterations; burn-in = 500,000; posterior thinning = 500; 3 chains). Model convergence was assessed using Gelman-Rubin and Geweke diagnostics (Gelman & Rubin, 1992; Geweke, 1992). Models were considered converged when no variables had a Gelman-Rubin diagnostic greater than 1.05 and where less than 5% of variables were outside the 95% confidence interval based on the Geweke diagnostic. Resulting posterior probability distributions were used to estimate the proportional contributions of the carbon sources to carnivorous fish food webs.

***CSIA trophic position estimation***

We calculated trophic positions (TP_CSIA_) for individual fish using the TP_CSIA_ equation (Chikaraishi et al., 2009):

$$Tophic Position \left( {TP}_{CSIA} \right)=1+ \frac{(\delta^{15}N_{TrophicAA}-\delta^{15}N_{SourceAA}-\beta)}{{TDF}_{Trophic-SourceAA}}$$

where δ^15^N_Trophic AA_ and δ^15^N_Source AA_ are the simple mean δ^15^N values of seven trophic (Ala, Val, Leu, Ile, Pro, Asx, Glx) and two source (Phe, Lys) AAs; TDF_Trophic AA–Source AA_ is the trophic discrimination factor reflecting changes in trophic and source AA δ^15^N values between diet and consumer (Avg_TrophicAA_–Avg_SourceAA_ = 5.5 ± 0.5 ‰ for a fish food webs) (Bradley et al., 2015); and β (beta) is the difference between trophic and source AA δ^15^N  values in the primary producer(s) at the base of the food web (Avg_TrophicAA_–Avg_SourceAA_ = 3.0 ± 2.4 ‰, non-vascular primary producer food web) (Ramirez et al., 2021). Errors were propagated using the *propagate* package in R (Spiess, 2018) using the aforementioned β and TDF value uncertainties and the error estimates resulting from triplicate sample injections for each amino acid for each fish.

Results

***δ^13^C_EAA_ fingerprinting: LDA vs. stable isotope mixing model***

The δ^13^C_EAA_ values of carbon source proxies differed for one or more amino acids, yielding strong separation among groups (Figure 2). The linear discriminant analysis (LDA) had 100% reclassification success rates for all carbon sources, confirming high differentiation in δ^13^C_EAA_ fingerprints. The first linear discriminant (LD_1_) explained 83.5% of the overall variation between groups, primarily driven by Ile and Leu (Table S6). The second linear discriminant (LD_2_) explained 13.9% of the variation, with variation primarily driven by Leu, Val, and Phe. The LDA classified 85% of carnivorous fish to the plankton group with > 75% probability (> 90% for 69 of 71 fish) and 15% of fish to the detritus group with > 60% probability (> 90% for 12 of 13 fish; Table S2). All *A. furca, L. bohar,* and *C. urodeta* were classified with plankton, whereas two of ten *C. melampygus*, two of fifteen *C. argus*, and nine of fifteen *L. fulvus* were classified with detritus. Detritus classification was not associated with human disturbance level. The bootstrapped LDA indicated that across the sampled carnivorous reef fish, carbon was derived primarily from plankton and detritus for 81% (95% CI: 48–90%) and 19% (95% CI: 10–52%) of fish, respectively. At the individual fish level, the bootstrapped LDA classification matched the traditional LDA classification for all fish, in most cases with a similar level of classification probability (Table S2). For both the normal and bootstrapped LDA, no individual fish were classified with the coral nor the epilithic algal matrix carbon sources.

The Bayesian stable isotope mixing models suggested higher variability in carbon source contribution to reef fish food webs than the LDA (Figure 2). There was general agreement between LDA and mixing models results that carbon sources supporting *A. furca*, *C. melampygus, L. bohar*, and *C. urodeta* were quite consistent among very low, medium, and very high disturbance sites. As in the bootstrapped LDA, mixing models identified plankton as the primary carbon source (Figures 2, 3 and Table S2). However, the mixing models also estimated non-trivial (12–31%) contributions of detrital carbon for these species. In contrast, the LDA and mixing model results were strongly divergent for *C. argus* and *L. fulvus*, with the mixing models suggesting detritus (53%) and coral (34%) were the primary carbon sources for *C. argus* and *L. fulvus* populations, respectively, as opposed to primarily plankton (and detritus: *L. fulvus*) as identified in the LDA. For individual *C. argus*, the mixing model identified detritus and plankton comprising 45–70% and 24–48% of assimilated carbon, respectively (Table S2). For individual *L. fulvus*, the mixing model identified contributions of 17–69% for coral, 18–55% for detritus, and 2–60% for plankton.

The divergent LDA and mixing model results for some species may be due in part to unavoidable limitations of the mixing model approach. First, this is likely a function of the way the LDA reports binary group membership relative to the more nuanced relative contribution data of the mixing model. Additionally, within stable isotope mixing models, strong correlations between contributions of sources can artificially inflate marginal uncertainty and broaden the estimated contributions of sources within food webs (Phillips et al., 2014). Here, we observed high detritus-coral and/or detritus-plankton correlations for a number of species (Table S7), including *C. melampygus* (detritus-plankton), *C. argus* (both) and *L. fulvus* (detritus-coral). This may have contributed to differences in mixing model results relative to the LDA and likely underpins the high uncertainty in posterior probability of source contributions for these species (Figure S2).

The generalist carnivore *L. fulvus* is a notable exception to the narrow carbon sourcing and resource use patterns observed for the other carnivorous fish species across the disturbance gradient, which may provide a case study for a life history strategy that may be the most buffered from disturbance. *L. fulvus* exhibited varied carbon source contributions among specific sampling sites as well as an ontogenetic change in resource use where they shifted from primarily coral- and detritally-supported food webs to primarily plankton-supported food webs. Similar ontogenetic resource shifts were observed for *L. fulvus* in southern Japan from mangroves to coral reefs (Nakamura et al., 2008) and *L. ehrenbergii* in the Red Sea from strong macroalgal (benthic) to planktonic (pelagic) sources (McMahon et al., 2012, 2016). Such plasticity in habitat associations, if not strict features of their life history, may make these taxa less vulnerable to disturbance-mediated changes in food web architecture than other carnivorous reef fishes.

***Bulk δ^13^C and δ^15^N value variation***

Although isotopic niche size and position generally did not differ among disturbance levels, we did observe variation in bulk SIA value for some species (Figure 3). For *A. furca,* bulk δ^15^N values were higher in medium vs. very low disturbance sites (Wilcoxon rank sum test, bonferroni adjusted *p*_M-VL_ = 0.008). For *C. urodeta*, bulk δ^15^N values were higher in medium and very high vs. very low disturbance sites (Wilcoxon rank sum test, bonferroni adjusted *p*_M-VL_ = 0.002 and *p*_VH-VL_ = 0.027). Lastly, for *L. fulvus*, bulk δ^13^C values were lower for medium vs. very low disturbance sites (Wilcoxon rank sum test, bonferroni adjusted *p*_M-VL_ = 0.01). Bulk δ^13^C and δ^15^N values did not differ among disturbance levels for all other pairwise comparisons (Kruskal-Wallis test, *p* > 0.05), including all data for *C. argus* and *L. bohar*.

***Source amino acid*** ***δ^15^N value variation***

Source amino acid δ^15^N values, a proxy for baseline N biogeochemical cycling, were highly variable among disturbance levels and species, and in some cases sampling sites (Figure S7). Phe δ^15^N values were both highly variable among individuals and distinctly higher for *C. argus* and *L. fulvus* than the other species. These patterns likely indicate variation in foraging microhabitat use across biogeochemical zones within the coral reef (Skinner et al., 2022). Within species, Phe δ^15^N values were notably higher at medium disturbance sites for *A. furca* and *C. melampygus* relative to the very low and very high disturbance sites. This indicates that the relatively high bulk δ^15^N values at medium disturbance levels for these species are driven primarily by baseline N isotope variation linked to local biogeochemical cycling (Figure 3), which on Kiritimati is influenced by both natural (e.g., island-wake upwelling (Walsh, 2011)) and anthropogenic (e.g., pollution) factors that vary spatially.


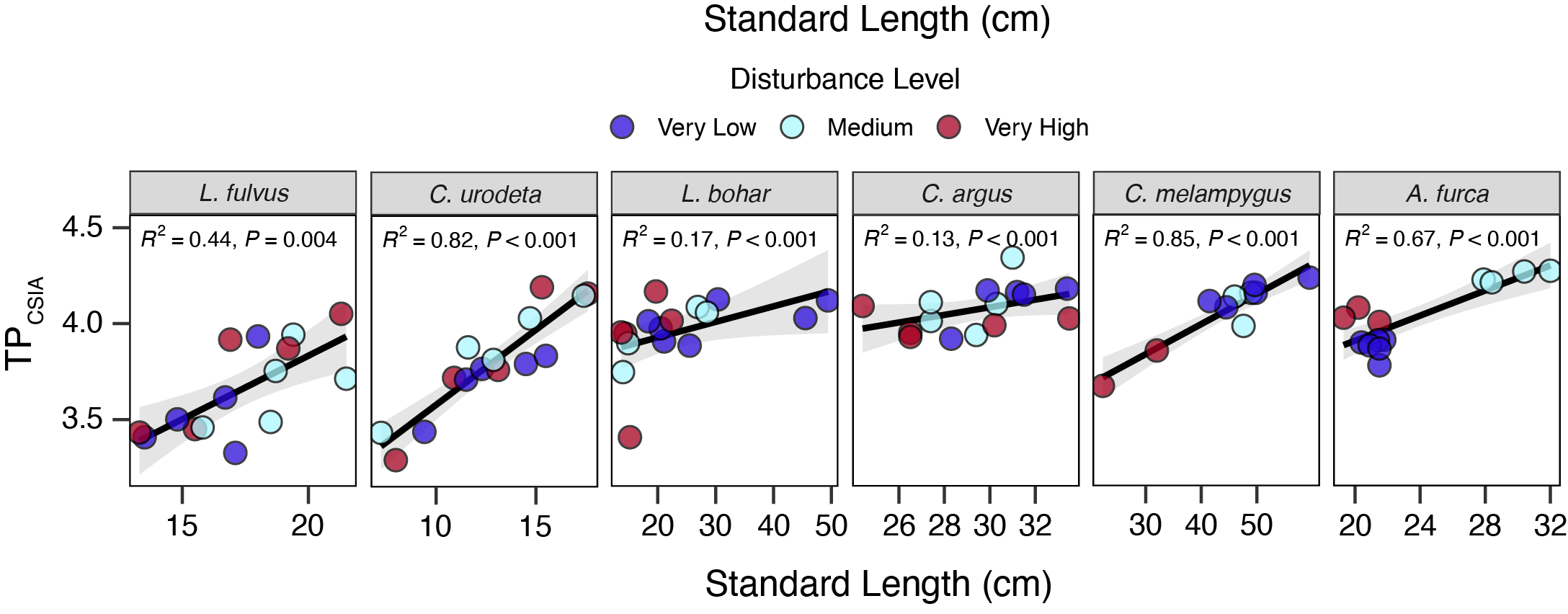


Figure S1. Body size-trophic position relationships for carnivorous reef fishes. General linear model fits of standard length versus uncorrected trophic position (TP_CSIA_) estimates for each sampled carnivorous reef fish species. Linear fits were used to size-correct TP_CSIA_ estimates.

**
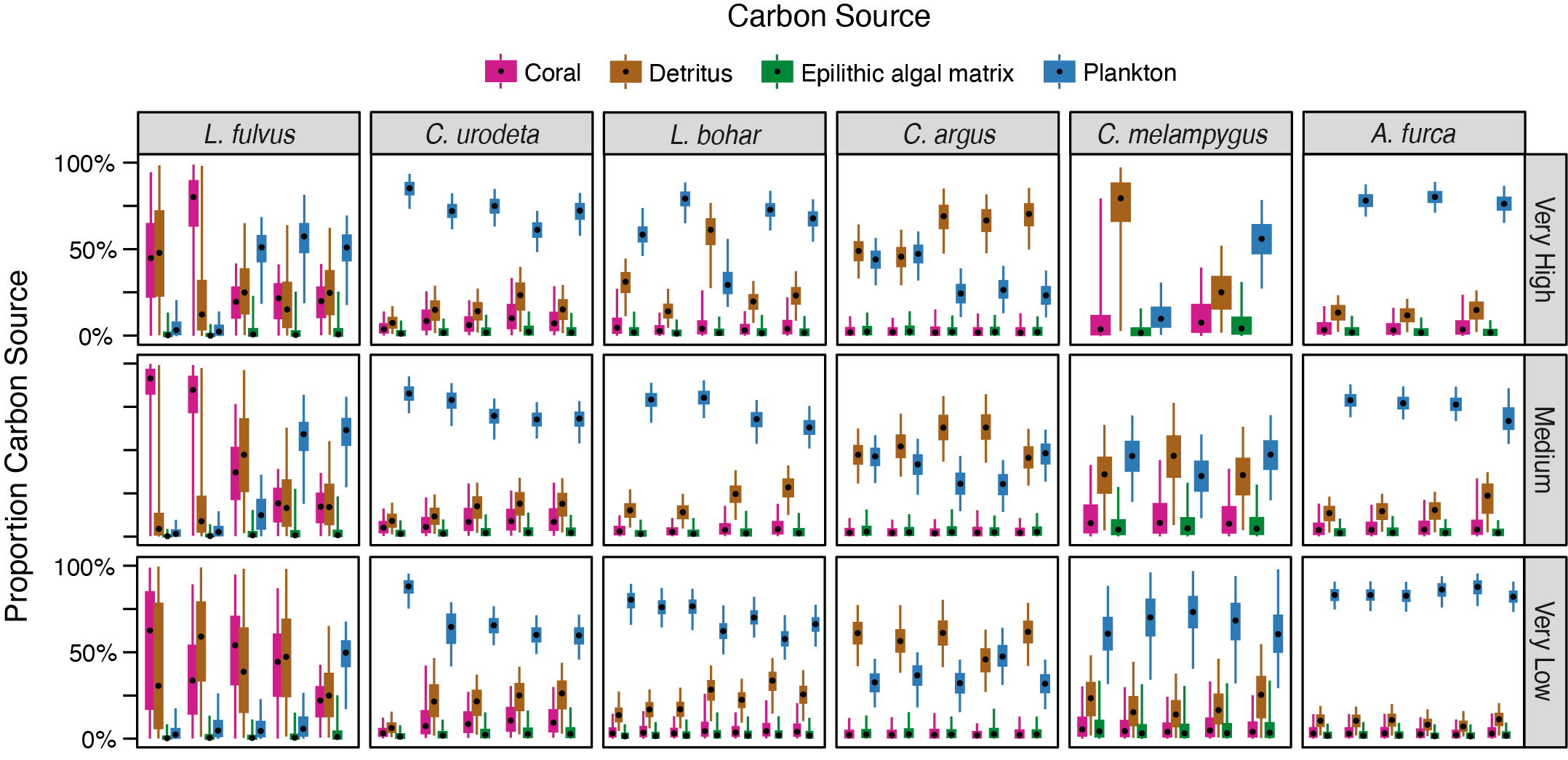
**

**Figure S2.** Proportional contributions of four basal production sources to individual carnivorous reef fish (clustered boxplots within panels) on coral reefs with different human disturbance levels. Source contributions were estimated using Bayesian stable isotope mixing models. Black points represent median values for individual fish. Clustered boxplots are ordered by increasing body size within species-specific panels. Generalist carnivores: LF = *L. fulvus,* CU = *C. urodeta*. Piscivores: LB = *L. bohar,* CA = *C. argus*, CM = *C. melampygus*, AF = *A. furca*.

**
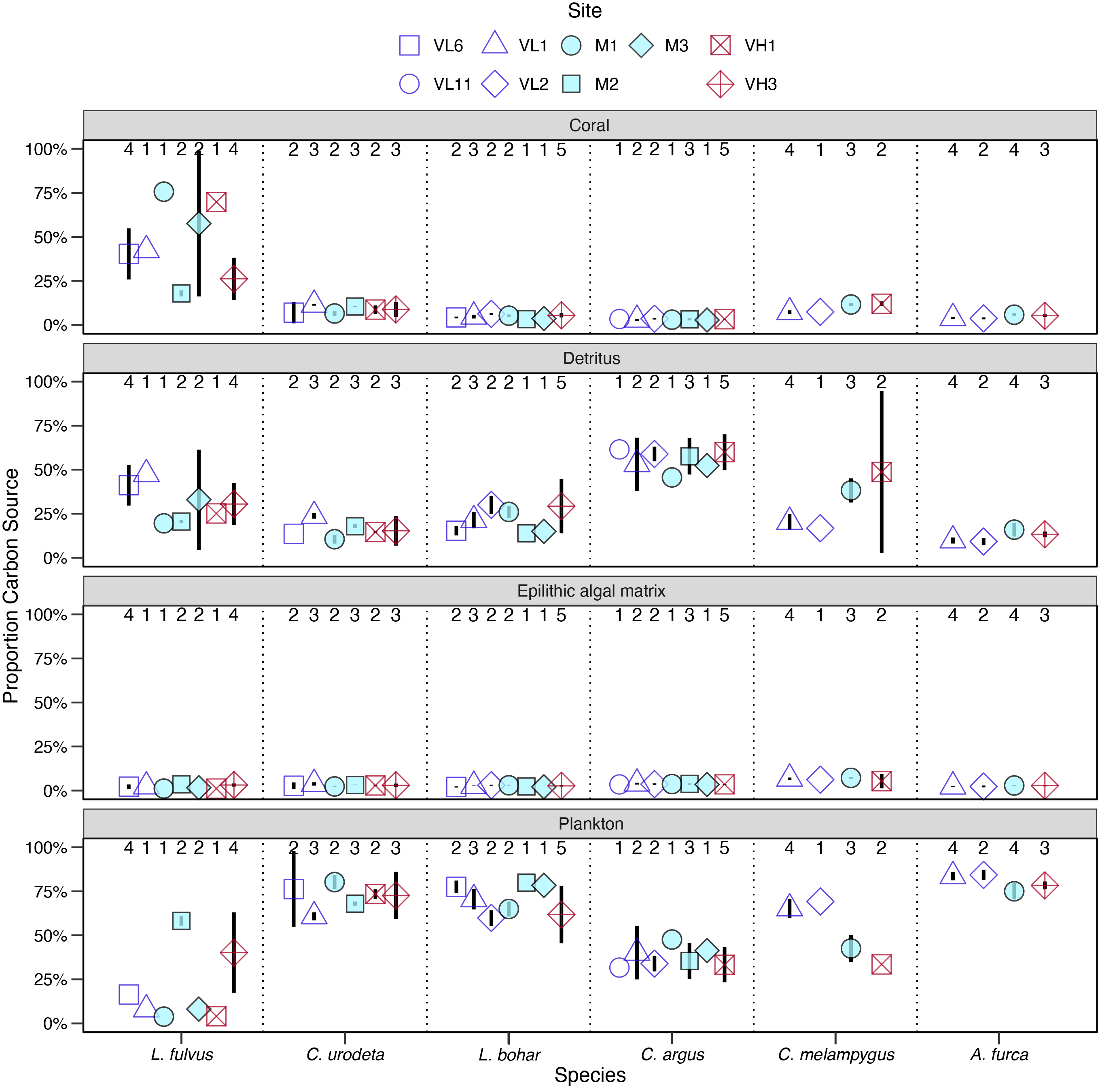
**

**Figure S3.** Sampling site-specific mean ± 95 % confidence intervals for contributions of four carbon source end-members (coral, detritus, epilithic algal matrix, plankton) to two generalist carnivore (*L. fulvus****,*** *C. urodeta*) and four piscivore (*L. bohar, C. argus*, *C. melampygus*, *A. furca*) reef fish on Kiritimati Atoll. Sample sizes are presented at the top of each panel. Means were estimated using Bayesian stable isotope mixing models parameterized with the δ^13^C values of six essential AAs (Thr, Val, Leu, Ile, Phe, Lys) for source end-members and consumers. Disturbance categories: VL = very low, M = medium, VH = very high.

**
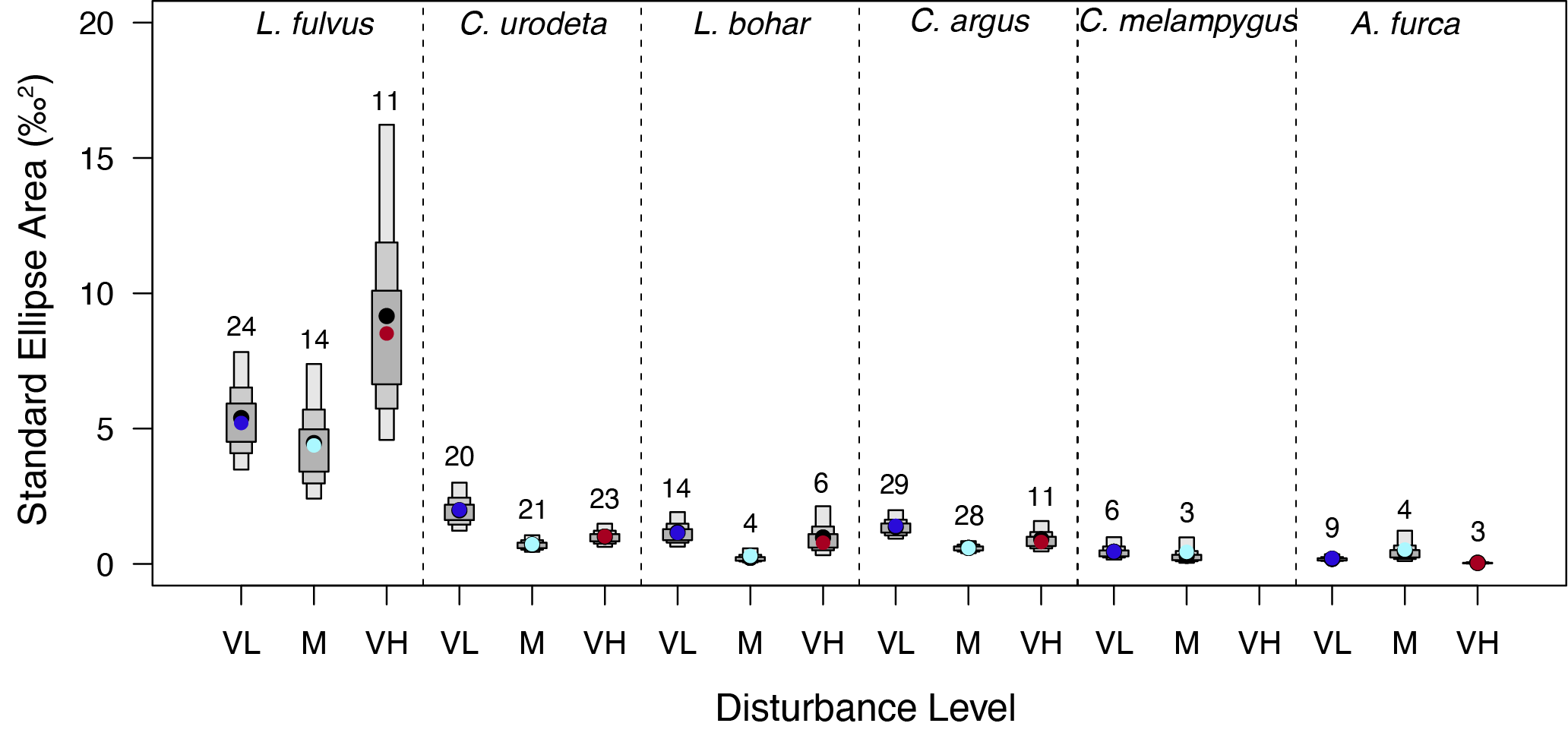
**

**Figure S4.** Isotopic niche sizes for carnivorous reef fishes. Bayesian Standard Ellipse Areas (SEA-B), where filled black circles represent the median SEA-B value, grey boxes represent the 50, 75, and 95 % credible intervals, and filled colored circles represent the mean SEAc values from Figure 4. SEAs are only reported for disturbance levels with N ≥ 3. VL = Very Low, M = Medium, VH = Very High.


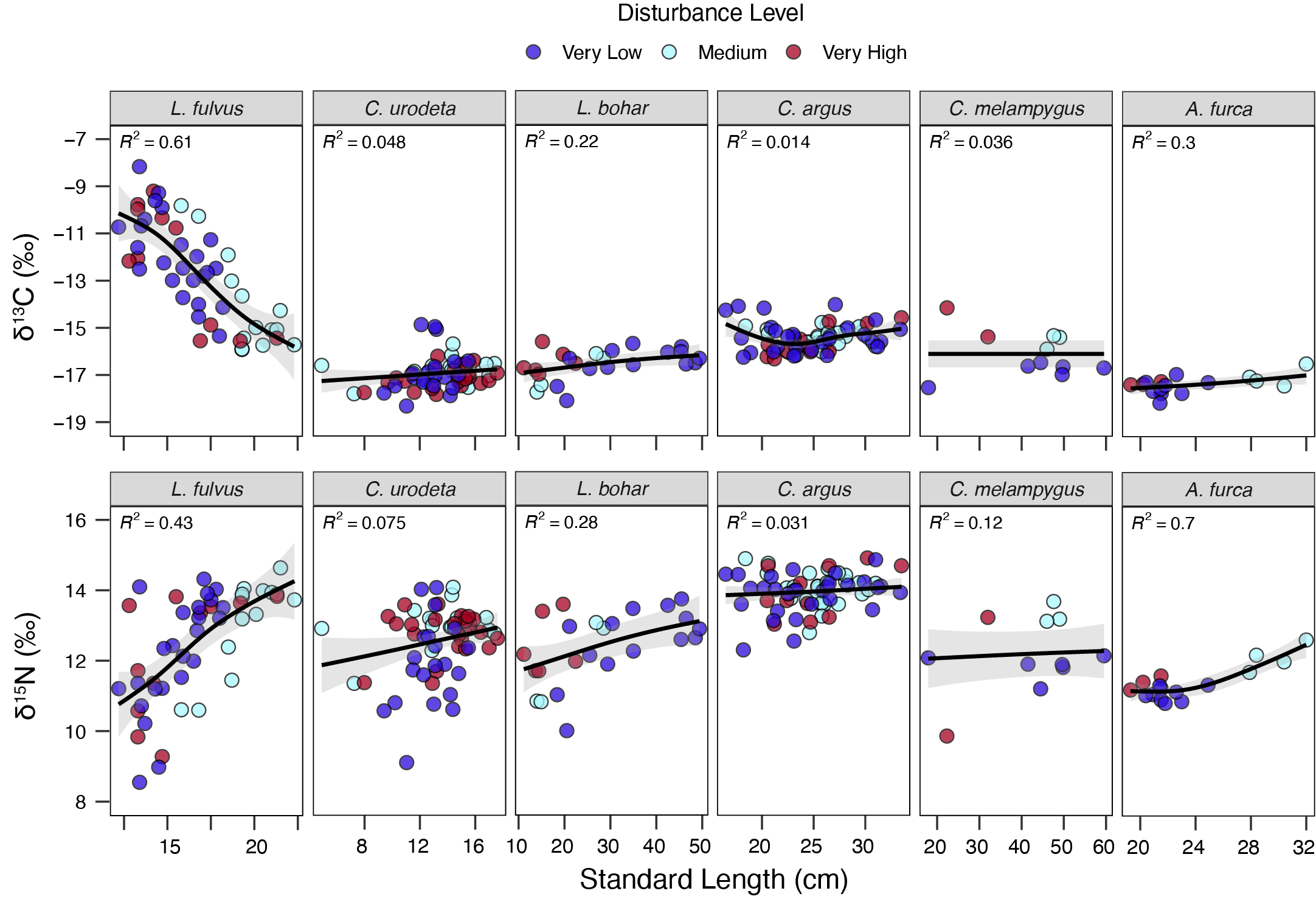


Figure S5. Body size-stable isotope relationships for carnivorous reef fishes. GAM fits of standard length versus stable carbon (δ^13^C) and nitrogen (δ^15^N) isotope values for each sampled carnivorous reef fish species.

**
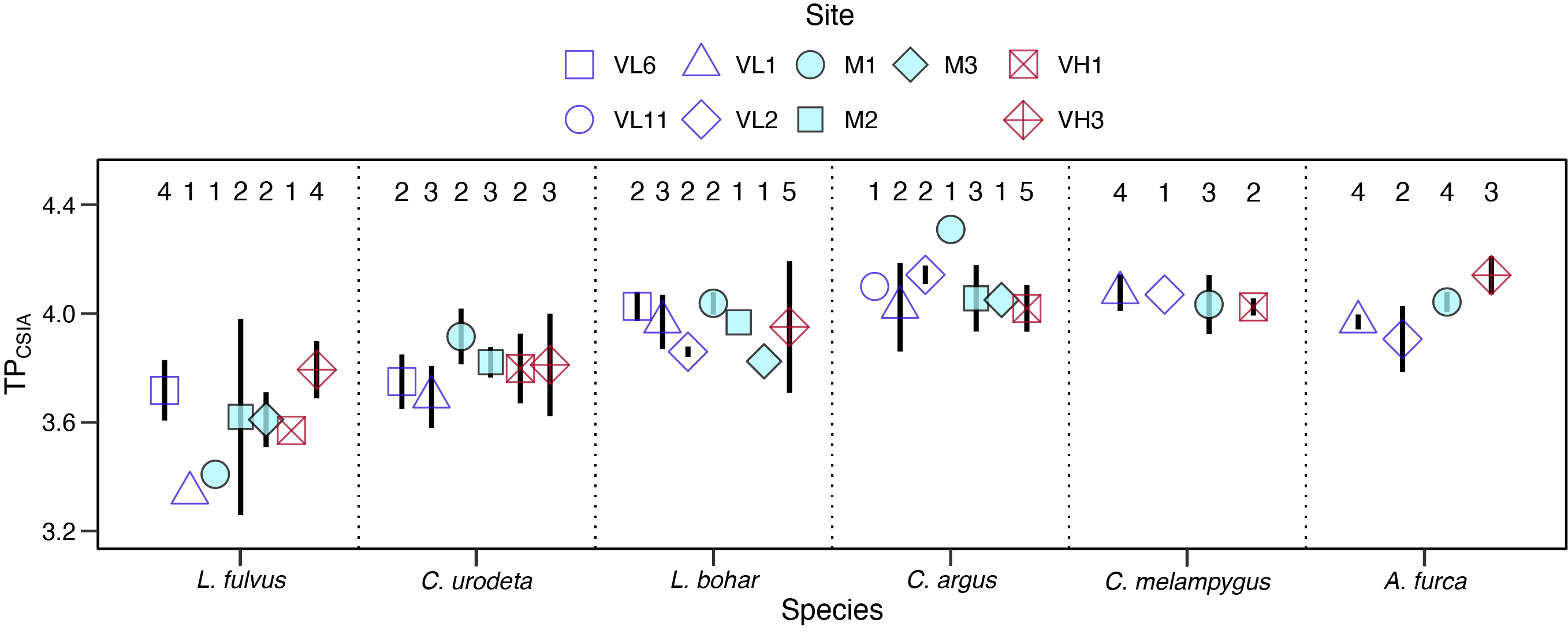
**

**Figure S6.** Sampling site-specific mean ± 95 % confidence intervals for size-corrected trophic position (TP_CSIA_) estimates by carnivorous reef fish species across the human disturbance gradient. Sample sizes are presented at the top of each panel. Disturbance categories: VL = very low, M = medium, VH = very high.


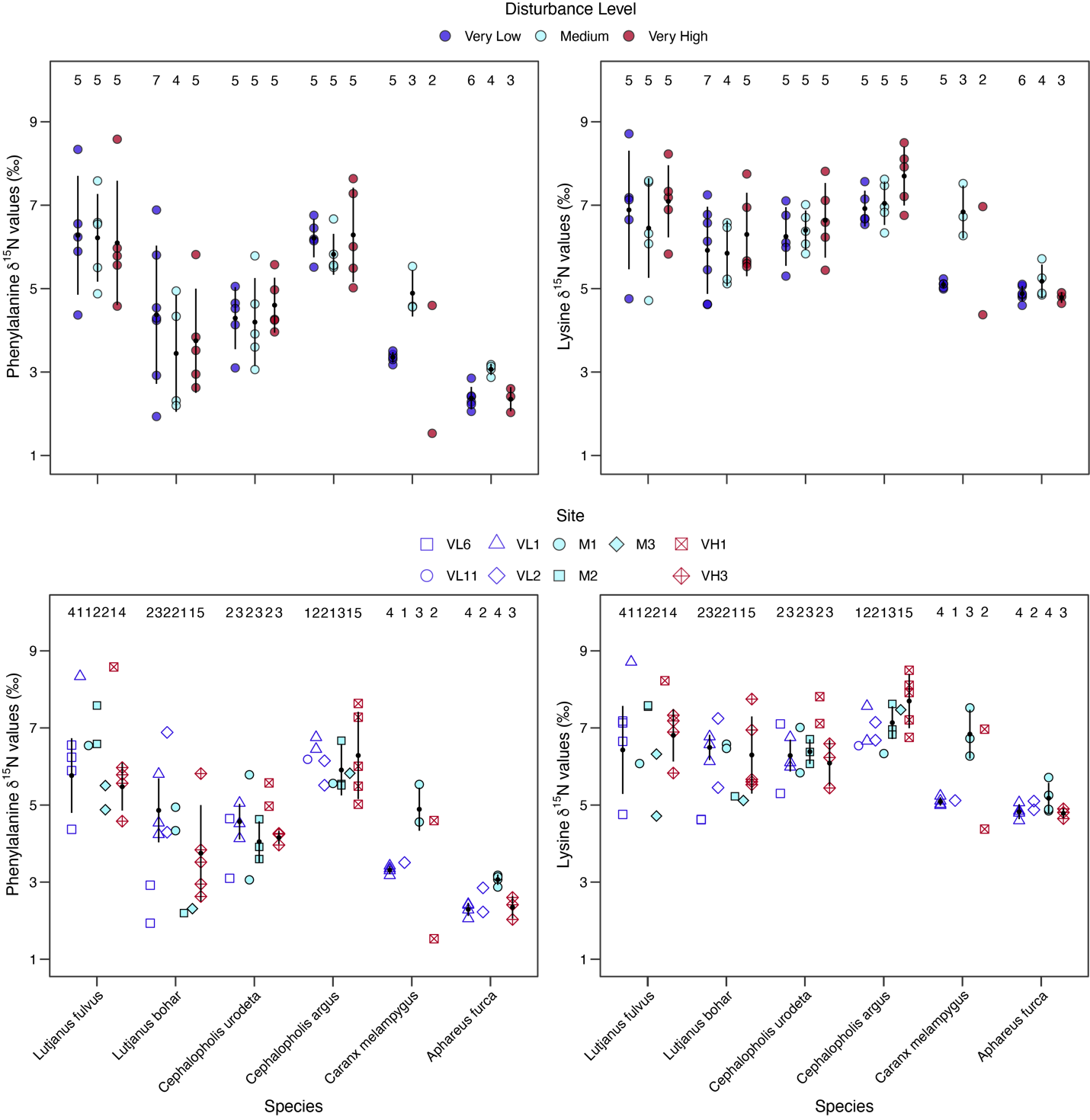


**Figure S7.** Source amino acid stable nitrogen isotope (δ^15^N) values by species, human disturbance level (upper panels), and sampling site (lower panels). Sample sizes are presented at the top of each panel. Where N ≥ 3, mean ± 1 SD is designated with a black point and error bars.**Table S1.** Characteristics of fish sampled via bulk stable isotope analysis (SIA) and compound-specific stable isotope analysis of amino acids (CSIA-AA).

| **Species/Disturbance Level** | **Bulk SIA** | | **CSIA-AA** | |
| --- | --- | --- | --- | --- |
|  | **N** | **Standard Length (cm)**  **Mean (Range)** | **N** | **Standard Length (cm)**  **Mean (Range)** |
|  |  |  |  |  |
| *Lutjanus fulvus* | 49 | 16.7 (12.2–22.3) | 15 | 17.3 (13.3–21.5) |
| Very Low | 24 | 15.6 (12.2–18.2) | 5 | 16.0 (13.5–18.0) |
| Medium | 14 | 19.6 (15.8–22.3) | 5 | 18.8 (15.8–21.5) |
| Very High | 11 | 15.6 (12.8–21.3) | 5 | 17.2 (13.3–21.3) |
| *Cephalopholis urodeta* | 64 | 13.5 (4.9–17.6) | 15 | 12.8 (7.2–17.6) |
| Very Low | 20 | 12.8 (9.4–15.5) | 5 | 12.6 (9.4–15.5) |
| Medium | 21 | 13.8 (4.9–17.4) | 5 | 12.8 (7.2–17.4) |
| Very High | 23 | 13.8 (8.0–17.6) | 5 | 13.0 (8.0–17.6) |
| *Lutjanus bohar* | 24 | 28.1 (11.1–49.4) | 16 | 23.8 (13.7–49.4) |
| Very Low | 14 | 35.2 (18.4–49.4) | 7 | 30.1 (18.4–49.4) |
| Medium | 4 | 21.1 (14.0–28.5) | 4 | 21.1 (14.0–28.5) |
| Very High | 6 | 16.1 (11.1–22.4) | 5 | 17.1 (13.7–22.4) |
| *Cephalopholis argus* | 68 | 23.6 (19.3–32.0) | 15 | 29.4 (24.4–33.5) |
| Very Low | 29 | 24.3 (16.5–33.4) | 5 | 30.9 (28.3–33.4) |
| Medium | 28 | 25.5 (18.4–31.0) | 5 | 29.1 (27.4–31.0) |
| Very High | 11 | 24.9 (20.5–33.5) | 5 | 28.2 (24.4–33.5) |
| *Caranx melampygus* | 11 | 41.8 (17.9–59.5) | 10 | 44.2 (22.3–59.5) |
| Very Low | 6 | 43.8 (17.9–59.5) | 5 | 49.0 (41.5–59.5) |
| Medium | 3 | 47.5 (46.0–49.0) | 3 | 47.5 (46.0–49.0) |
| Very High | 2 | 27.2 (22.3–32.0) | 2 | 27.2 (22.3–32.0) |
| *Aphareus furca* | 16 | 23.6 (19.3–32.0) | 13 | 23.6 (19.3–32.) |
| Very Low | 9 | 22.0 (20.4–24.9) | 6 | 21.3 (20.4–21.8) |
| Medium | 4 | 29.7 (27.9–32.0) | 4 | 29.7 (27.9–32.0) |
| Very High | 3 | 20.3 (19.3–21.5) | 3 | 20.3 (19.3–21.5) |

Table S2. Posterior probabilities of classification of carnivorous fish via simple LDA, bootstrapped LDA (LDA_boot_), and Bayesian Stable Isotope Mixing Model (SIMM). Fish are ordered by increasing body size within disturbance levels. Bold, highlighted cells identify proportional contributions ≥ 0.50.

| **Fish Code** | **Species** | **Disturbance Level** | **Standard Length (cm)** | **Coral** | | | **Detritus** | | | **Epilithic Algal Matrix** | | | **Plankton** | | |
| --- | --- | --- | --- | --- | --- | --- | --- | --- | --- | --- | --- | --- | --- | --- | --- |
|  |  |  |  | **LDA** | **LDA_boot_** | **SIMM** | **LDA** | **LDA_boot_** | **SIMM** | **LDA** | **LDA_boot_** | **SIMM** | **LDA** | **LDA_boot_** | **SIMM** |
| AF13 | *Aphareus furca* | Very Low | 20.4 | 0.00 | 0.00 | 0.04 | 0.00 | 0.00 | 0.10 | 0.00 | 0.00 | 0.02 | **1.00** | **1.00** | **0.83** |
| AF8 | *Aphareus furca* | Very Low | 20.9 | 0.00 | 0.00 | 0.04 | 0.00 | 0.00 | 0.10 | 0.00 | 0.00 | 0.03 | **1.00** | **1.00** | **0.83** |
| AF12 | *Aphareus furca* | Very Low | 21.4 | 0.00 | 0.00 | 0.04 | 0.00 | 0.00 | 0.11 | 0.00 | 0.00 | 0.03 | **1.00** | **1.00** | **0.82** |
| AF7 | *Aphareus furca* | Very Low | 21.5 | 0.00 | 0.00 | 0.04 | 0.00 | 0.00 | 0.08 | 0.00 | 0.00 | 0.02 | **1.00** | **1.00** | **0.86** |
| AF9 | *Aphareus furca* | Very Low | 21.5 | 0.00 | 0.00 | 0.03 | 0.00 | 0.00 | 0.07 | 0.00 | 0.00 | 0.02 | **1.00** | **1.00** | **0.87** |
| AF11 | *Aphareus furca* | Very Low | 21.8 | 0.00 | 0.00 | 0.04 | 0.00 | 0.00 | 0.11 | 0.00 | 0.00 | 0.03 | **1.00** | **1.00** | **0.82** |
| AF4 | *Aphareus furca* | Medium | 27.9 | 0.00 | 0.00 | 0.05 | 0.00 | 0.00 | 0.13 | 0.00 | 0.00 | 0.03 | **1.00** | **1.00** | **0.79** |
| AF5 | *Aphareus furca* | Medium | 28.4 | 0.00 | 0.00 | 0.05 | 0.00 | 0.00 | 0.14 | 0.00 | 0.00 | 0.03 | **1.00** | **1.00** | **0.77** |
| AF6 | *Aphareus furca* | Medium | 30.4 | 0.00 | 0.00 | 0.06 | 0.00 | 0.00 | 0.15 | 0.00 | 0.00 | 0.03 | **1.00** | **1.00** | **0.76** |
| AF10 | *Aphareus furca* | Medium | 32.0 | 0.00 | 0.00 | 0.07 | 0.00 | 0.02 | 0.22 | 0.00 | 0.00 | 0.03 | **1.00** | **0.98** | **0.68** |
| AF3 | *Aphareus furca* | Very High | 19.3 | 0.00 | 0.00 | 0.05 | 0.00 | 0.00 | 0.13 | 0.00 | 0.00 | 0.03 | **1.00** | **1.00** | **0.78** |
| AF2 | *Aphareus furca* | Very High | 20.2 | 0.00 | 0.00 | 0.05 | 0.00 | 0.00 | 0.12 | 0.00 | 0.00 | 0.03 | **1.00** | **1.00** | **0.80** |
| AF1 | *Aphareus furca* | Very High | 21.5 | 0.00 | 0.00 | 0.06 | 0.00 | 0.00 | 0.15 | 0.00 | 0.00 | 0.03 | **1.00** | **1.00** | **0.77** |
| CM8 | *Caranx melampygus* | Very Low | 41.5 | 0.00 | 0.00 | 0.08 | 0.00 | 0.09 | 0.23 | 0.00 | 0.00 | 0.08 | **1.00** | **0.91** | **0.61** |
| CM6 | *Caranx melampygus* | Very Low | 44.5 | 0.00 | 0.00 | 0.08 | 0.00 | 0.02 | 0.17 | 0.00 | 0.00 | 0.06 | **1.00** | **0.98** | **0.69** |
| CM9 | *Caranx melampygus* | Very Low | 49.6 | 0.00 | 0.00 | 0.06 | 0.00 | 0.02 | 0.15 | 0.00 | 0.00 | 0.06 | **1.00** | **0.99** | **0.72** |
| CM7 | *Caranx melampygus* | Very Low | 49.8 | 0.00 | 0.00 | 0.08 | 0.00 | 0.03 | 0.18 | 0.00 | 0.00 | 0.07 | **1.00** | **0.97** | **0.67** |
| CM10 | *Caranx melampygus* | Very Low | 59.5 | 0.00 | 0.00 | 0.07 | 0.00 | 0.04 | 0.25 | 0.00 | 0.00 | 0.07 | **1.00** | **0.96** | **0.61** |
| CM2 | *Caranx melampygus* | Medium | 46.0 | 0.00 | 0.00 | 0.12 | 0.00 | 0.13 | 0.35 | 0.00 | 0.00 | 0.07 | **1.00** | **0.87** | 0.46 |
| CM3 | *Caranx melampygus* | Medium | 47.6 | 0.00 | 0.00 | 0.12 | **0.92** | **0.67** | 0.45 | 0.00 | 0.00 | 0.08 | 0.08 | 0.33 | 0.35 |
| CM1 | *Caranx melampygus* | Medium | 49.0 | 0.00 | 0.00 | 0.11 | 0.04 | 0.23 | 0.35 | 0.00 | 0.00 | 0.08 | **0.96** | **0.77** | 0.47 |
| CM4 | *Caranx melampygus* | Very High | 22.3 | 0.00 | 0.00 | 0.13 | **1.00** | **0.82** | **0.72** | 0.00 | 0.00 | 0.03 | 0.00 | 0.18 | 0.12 |
| CM5 | *Caranx melampygus* | Very High | 32.0 | 0.00 | 0.00 | 0.12 | 0.00 | 0.07 | 0.25 | 0.00 | 0.00 | 0.08 | **1.00** | **0.93** | **0.56** |
| CA8 | *Cephalopholis argus* | Very Low | 28.3 | 0.00 | 0.00 | 0.03 | 0.11 | 0.35 | **0.61** | 0.00 | 0.00 | 0.04 | **0.89** | **0.65** | 0.32 |
| CA13 | *Cephalopholis argus* | Very Low | 29.9 | 0.00 | 0.00 | 0.03 | 0.00 | 0.16 | **0.57** | 0.00 | 0.00 | 0.04 | **1.00** | **0.84** | 0.36 |
| CA11 | *Cephalopholis argus* | Very Low | 31.2 | 0.00 | 0.00 | 0.04 | 0.00 | 0.13 | **0.61** | 0.00 | 0.00 | 0.04 | **1.00** | **0.87** | 0.32 |
| CA7 | *Cephalopholis argus* | Very Low | 31.5 | 0.00 | 0.00 | 0.03 | 0.01 | 0.21 | 0.45 | 0.00 | 0.00 | 0.04 | **0.99** | **0.79** | 0.48 |
| CA9 | *Cephalopholis argus* | Very Low | 33.4 | 0.00 | 0.00 | 0.03 | 0.01 | 0.25 | **0.62** | 0.00 | 0.00 | 0.04 | **0.99** | **0.75** | 0.32 |
| CA3 | *Cephalopholis argus* | Medium | 27.4 | 0.00 | 0.00 | 0.03 | 0.00 | 0.09 | 0.47 | 0.00 | 0.00 | 0.04 | **1.00** | **0.91** | 0.46 |
| CA10 | *Cephalopholis argus* | Medium | 27.4 | 0.00 | 0.00 | 0.03 | 0.00 | 0.07 | **0.52** | 0.00 | 0.00 | 0.04 | **1.00** | **0.93** | 0.41 |
| CA4 | *Cephalopholis argus* | Medium | 29.4 | 0.00 | 0.00 | 0.03 | 0.25 | 0.44 | **0.63** | 0.00 | 0.00 | 0.04 | **0.75** | **0.56** | 0.31 |
| CA12 | *Cephalopholis argus* | Medium | 30.3 | 0.00 | 0.00 | 0.04 | 0.00 | 0.14 | **0.63** | 0.00 | 0.00 | 0.03 | **1.00** | **0.86** | 0.30 |
| CA1 | *Cephalopholis argus* | Medium | 31.0 | 0.00 | 0.00 | 0.03 | 0.00 | 0.03 | 0.46 | 0.00 | 0.00 | 0.04 | **1.00** | **0.97** | 0.48 |
| CA2 | *Cephalopholis argus* | Very High | 24.4 | 0.00 | 0.00 | 0.03 | 0.00 | 0.04 | 0.49 | 0.00 | 0.00 | 0.04 | **1.00** | **0.97** | 0.44 |
| CA14 | *Cephalopholis argus* | Very High | 26.5 | 0.00 | 0.00 | 0.03 | 0.00 | 0.02 | 0.46 | 0.00 | 0.00 | 0.04 | **1.00** | **0.98** | 0.47 |
| CA15 | *Cephalopholis argus* | Very High | 26.5 | 0.00 | 0.00 | 0.04 | 0.08 | 0.35 | **0.69** | 0.00 | 0.00 | 0.03 | **0.92** | **0.65** | 0.25 |
| CA6 | *Cephalopholis argus* | Very High | 30.2 | 0.00 | 0.00 | 0.03 | **0.64** | **0.52** | **0.66** | 0.00 | 0.00 | 0.03 | 0.36 | 0.48 | 0.27 |
| CA5 | *Cephalopholis argus* | Very High | 33.5 | 0.00 | 0.00 | 0.03 | **0.96** | **0.67** | **0.70** | 0.00 | 0.00 | 0.03 | 0.04 | 0.33 | 0.24 |
| LB5 | *Lutjanus bohar* | Very Low | 18.4 | 0.00 | 0.00 | 0.04 | 0.00 | 0.00 | 0.14 | 0.00 | 0.00 | 0.02 | **1.00** | **1.00** | **0.80** |
| LB4 | *Lutjanus bohar* | Very Low | 20.5 | 0.00 | 0.00 | 0.05 | 0.00 | 0.00 | 0.17 | 0.00 | 0.00 | 0.02 | **1.00** | **1.00** | **0.76** |
| LB13 | *Lutjanus bohar* | Very Low | 21.1 | 0.00 | 0.00 | 0.04 | 0.00 | 0.00 | 0.17 | 0.00 | 0.00 | 0.03 | **1.00** | **1.00** | **0.76** |
| LB9 | *Lutjanus bohar* | Very Low | 25.5 | 0.00 | 0.00 | 0.07 | 0.00 | 0.05 | 0.28 | 0.00 | 0.00 | 0.03 | **1.00** | **0.95** | **0.62** |
| LB14 | *Lutjanus bohar* | Very Low | 30.4 | 0.00 | 0.00 | 0.05 | 0.00 | 0.00 | 0.22 | 0.00 | 0.00 | 0.03 | **1.00** | **1.00** | **0.70** |
| LB16 | *Lutjanus bohar* | Very Low | 45.5 | 0.00 | 0.00 | 0.06 | 0.00 | 0.06 | 0.33 | 0.00 | 0.00 | 0.03 | **1.00** | **0.94** | **0.58** |
| LB17 | *Lutjanus bohar* | Very Low | 49.4 | 0.00 | 0.00 | 0.06 | 0.00 | 0.03 | 0.25 | 0.00 | 0.00 | 0.03 | **1.00** | **0.98** | **0.66** |
| LB1 | *Lutjanus bohar* | Medium | 14.0 | 0.00 | 0.00 | 0.04 | 0.00 | 0.00 | 0.15 | 0.00 | 0.00 | 0.02 | **1.00** | **1.00** | **0.79** |
| LB2 | *Lutjanus bohar* | Medium | 14.9 | 0.00 | 0.00 | 0.03 | 0.00 | 0.00 | 0.14 | 0.00 | 0.00 | 0.03 | **1.00** | **1.00** | **0.80** |
| LB10 | *Lutjanus bohar* | Medium | 26.9 | 0.00 | 0.00 | 0.05 | 0.00 | 0.03 | 0.25 | 0.00 | 0.00 | 0.03 | **1.00** | **0.97** | **0.67** |
| LB3 | *Lutjanus bohar* | Medium | 28.5 | 0.00 | 0.00 | 0.06 | 0.00 | 0.01 | 0.28 | 0.00 | 0.00 | 0.03 | **1.00** | **0.99** | **0.63** |
| LB6 | *Lutjanus bohar* | Very High | 13.7 | 0.00 | 0.00 | 0.07 | 0.00 | 0.03 | 0.30 | 0.00 | 0.00 | 0.03 | **1.00** | **0.97** | **0.59** |
| LB11 | *Lutjanus bohar* | Very High | 14.4 | 0.00 | 0.00 | 0.04 | 0.00 | 0.00 | 0.15 | 0.00 | 0.00 | 0.02 | **1.00** | **1.00** | **0.79** |
| LB8 | *Lutjanus bohar* | Very High | 15.2 | 0.00 | 0.00 | 0.07 | 0.07 | 0.30 | **0.59** | 0.00 | 0.00 | 0.03 | **0.93** | **0.70** | 0.32 |
| LB12 | *Lutjanus bohar* | Very High | 19.7 | 0.00 | 0.00 | 0.05 | 0.00 | 0.00 | 0.20 | 0.00 | 0.00 | 0.03 | **1.00** | **1.00** | **0.73** |
| LB7 | *Lutjanus bohar* | Very High | 22.4 | 0.00 | 0.00 | 0.06 | 0.00 | 0.01 | 0.23 | 0.00 | 0.00 | 0.03 | **1.00** | **0.99** | **0.68** |
| CU1 | *Cephalopholis urodeta* | Very Low | 9.4 | 0.00 | 0.00 | 0.04 | 0.00 | 0.00 | 0.07 | 0.00 | 0.00 | 0.02 | **1.00** | **1.00** | **0.87** |
| CU15 | *Cephalopholis urodeta* | Very Low | 11.5 | 0.00 | 0.00 | 0.12 | 0.00 | 0.03 | 0.22 | 0.00 | 0.00 | 0.03 | **1.00** | **0.97** | **0.63** |
| CU2 | *Cephalopholis urodeta* | Very Low | 12.3 | 0.00 | 0.00 | 0.10 | 0.00 | 0.13 | 0.21 | 0.00 | 0.00 | 0.04 | **1.00** | **0.87** | **0.65** |
| CU12 | *Cephalopholis urodeta* | Very Low | 14.5 | 0.00 | 0.00 | 0.12 | 0.00 | 0.05 | 0.24 | 0.00 | 0.00 | 0.04 | **1.00** | **0.95** | **0.60** |
| CU5 | *Cephalopholis urodeta* | Very Low | 15.5 | 0.00 | 0.00 | 0.11 | 0.01 | 0.10 | 0.25 | 0.00 | 0.00 | 0.04 | **0.99** | **0.90** | **0.59** |
| CU7 | *Cephalopholis urodeta* | Medium | 7.2 | 0.00 | 0.00 | 0.06 | 0.00 | 0.00 | 0.09 | 0.00 | 0.00 | 0.02 | **1.00** | **1.00** | **0.82** |
| CU13 | *Cephalopholis urodeta* | Medium | 11.6 | 0.00 | 0.00 | 0.07 | 0.00 | 0.02 | 0.12 | 0.00 | 0.00 | 0.03 | **1.00** | **0.98** | **0.78** |
| CU8 | *Cephalopholis urodeta* | Medium | 12.9 | 0.00 | 0.00 | 0.11 | 0.00 | 0.01 | 0.17 | 0.00 | 0.00 | 0.03 | **1.00** | **1.00** | **0.69** |
| CU10 | *Cephalopholis urodeta* | Medium | 14.7 | 0.00 | 0.00 | 0.10 | 0.00 | 0.00 | 0.19 | 0.00 | 0.00 | 0.04 | **1.00** | **1.00** | **0.68** |
| CU9 | *Cephalopholis urodeta* | Medium | 17.4 | 0.00 | 0.00 | 0.11 | 0.00 | 0.01 | 0.18 | 0.00 | 0.00 | 0.03 | **1.00** | **0.99** | **0.68** |
| CU3 | *Cephalopholis urodeta* | Very High | 8.0 | 0.00 | 0.00 | 0.05 | 0.00 | 0.00 | 0.08 | 0.00 | 0.00 | 0.02 | **1.00** | **1.00** | **0.85** |
| CU14 | *Cephalopholis urodeta* | Very High | 10.9 | 0.00 | 0.00 | 0.10 | 0.00 | 0.00 | 0.15 | 0.00 | 0.00 | 0.03 | **1.00** | **1.00** | **0.72** |
| CU4 | *Cephalopholis urodeta* | Very High | 13.1 | 0.00 | 0.00 | 0.08 | 0.00 | 0.01 | 0.14 | 0.00 | 0.00 | 0.03 | **1.00** | **0.99** | **0.75** |
| CU11 | *Cephalopholis urodeta* | Very High | 15.3 | 0.00 | 0.00 | 0.12 | 0.00 | 0.03 | 0.23 | 0.00 | 0.00 | 0.04 | **1.00** | **0.97** | **0.61** |
| CU6 | *Cephalopholis urodeta* | Very High | 17.6 | 0.00 | 0.00 | 0.10 | 0.00 | 0.00 | 0.15 | 0.00 | 0.00 | 0.03 | **1.00** | **1.00** | **0.72** |
| LF1 | *Lutjanus fulvus* | Very Low | 13.5 | 0.00 | 0.00 | **0.54** | **1.00** | **0.98** | 0.41 | 0.00 | 0.02 | 0.01 | 0.00 | 0.00 | 0.04 |
| LF15 | *Lutjanus fulvus* | Very Low | 14.8 | 0.00 | 0.00 | 0.36 | **1.00** | **1.00** | **0.55** | 0.00 | 0.00 | 0.02 | 0.00 | 0.00 | 0.07 |
| LF2 | *Lutjanus fulvus* | Very Low | 16.7 | 0.00 | 0.00 | 0.50 | **1.00** | **1.00** | 0.42 | 0.00 | 0.00 | 0.02 | 0.00 | 0.00 | 0.07 |
| LF12 | *Lutjanus fulvus* | Very Low | 17.1 | 0.00 | 0.00 | 0.42 | **1.00** | **1.00** | 0.47 | 0.00 | 0.00 | 0.02 | 0.00 | 0.00 | 0.08 |
| LF6 | *Lutjanus fulvus* | Very Low | 18.0 | 0.00 | 0.00 | 0.22 | 0.00 | 0.07 | 0.27 | 0.00 | 0.00 | 0.04 | **1.00** | **0.93** | 0.48 |
| LF7 | *Lutjanus fulvus* | Medium | 15.8 | 0.00 | 0.00 | **0.79** | **1.00** | **1.00** | 0.18 | 0.00 | 0.00 | 0.01 | 0.00 | 0.00 | 0.02 |
| LF8 | *Lutjanus fulvus* | Medium | 18.5 | 0.00 | 0.00 | **0.76** | **1.00** | **0.99** | 0.20 | 0.00 | 0.00 | 0.01 | 0.00 | 0.01 | 0.04 |
| LF13 | *Lutjanus fulvus* | Medium | 18.7 | 0.00 | 0.00 | 0.36 | **1.00** | **0.88** | 0.47 | 0.00 | 0.00 | 0.02 | 0.00 | 0.13 | 0.14 |
| LF10 | *Lutjanus fulvus* | Medium | 19.4 | 0.00 | 0.00 | 0.19 | 0.00 | 0.06 | 0.21 | 0.00 | 0.00 | 0.04 | **1.00** | **0.94** | **0.57** |
| LF9 | *Lutjanus fulvus* | Medium | 21.5 | 0.00 | 0.00 | 0.17 | 0.00 | 0.03 | 0.20 | 0.00 | 0.00 | 0.04 | **1.00** | **0.97** | **0.60** |
| LF3 | *Lutjanus fulvus* | Very High | 13.3 | 0.00 | 0.00 | 0.44 | **1.00** | **1.00** | 0.48 | 0.00 | 0.00 | 0.02 | 0.00 | 0.00 | 0.06 |
| LF14 | *Lutjanus fulvus* | Very High | 15.5 | 0.00 | 0.00 | **0.70** | **1.00** | **1.00** | 0.25 | 0.00 | 0.00 | 0.01 | 0.00 | 0.00 | 0.04 |
| LF4 | *Lutjanus fulvus* | Very High | 16.9 | 0.00 | 0.00 | 0.20 | 0.01 | 0.10 | 0.27 | 0.00 | 0.00 | 0.04 | **0.99** | **0.90** | **0.50** |
| LF11 | *Lutjanus fulvus* | Very High | 19.2 | 0.00 | 0.00 | 0.21 | 0.00 | 0.04 | 0.20 | 0.00 | 0.00 | 0.03 | **1.00** | **0.96** | **0.56** |
| LF5 | *Lutjanus fulvus* | Very High | 21.3 | 0.00 | 0.00 | 0.20 | 0.01 | 0.12 | 0.26 | 0.00 | 0.00 | 0.04 | **0.99** | **0.88** | **0.50** |

Table S3. Output from SIBER models comparing baseline corrected bulk tissue stable carbon (δ^13^C) and nitrogen (δ^15^N) isotope values for carnivorous reef fish by human disturbance level. SEAc = Standard Ellipse Areas corrected for sample size, SEA-B = Bayesian Standard Ellipse Areas. SEAs are only reported for disturbance levels with N ≥ 3.

|  |  | **SEA-B** | | |
| --- | --- | --- | --- | --- |
| **Species/Disturbance Level** | **SEAc** | **Mode** | **95% CI (lower)** | **95% CI (upper)** |
| *Lutjanus fulvus* |  |  |  |  |
| Very Low (n = 24) | 5.21 | 5.24 | 3.37 | 7.84 |
| Medium (n = 14) | 4.38 | 3.92 | 2.38 | 7.32 |
| Very High (n = 11) | 8.51 | 8.10 | 4.55 | 15.71 |
| *Cephalopholis urodeta* |  |  |  |  |
| Very Low (n = 14) | 2.00 | 1.87 | 1.19 | 3.02 |
| Medium (n = 4) | 0.73 | 0.68 | 0.44 | 1.07 |
| Very High (n = 6) | 1.02 | 0.98 | 0.60 | 1.46 |
| *Lutjanus bohar* |  |  |  |  |
| Very Low (n = 20) | 1.15 | 1.11 | 0.61 | 1.91 |
| Medium (n = 21) | 0.30 | 0.16 | 0.06 | 0.61 |
| Very High (n = 23) | 0.78 | 0.80 | 0.33 | 2.13 |
| *Cephalopholis argus* |  |  |  |  |
| Very Low (n = 29) | 1.41 | 1.31 | 0.92 | 1.95 |
| Medium (n = 28) | 0.59 | 0.56 | 0.39 | 0.84 |
| Very High (n = 11) | 0.81 | 0.83 | 0.41 | 1.60 |
| *Caranx melampygus* |  |  |  |  |
| Very Low (n = 6) | 0.46 | 0.35 | 0.17 | 0.97 |
| Medium (n = 3) | 0.45 | 0.21 | 0.06 | 0.93 |
| Very High (n = 2) | NA | NA | NA | NA |
| *Aphareus furca* |  |  |  |  |
| Very Low (n = 9) | 0.21 | 0.16 | 0.09 | 0.35 |
| Medium (n = 4) | 0.53 | 0.33 | 0.11 | 1.22 |
| Very High (n = 3) | 0.04 | 0.03 | 0.01 | 0.13 |

Table S4. Comparisons of isotopic niche size among human disturbance levels. Probability that Bayesian Standard Ellipse Area (SEA-B) posterior distribution ellipse size (i.e., niche size) differs between disturbance levels. Differences were considered significant if ≥ 95% of posterior draws (10^4^) were smaller or larger than the other (highlighted cells). VL = Very Low, M = Medium, VH = Very High.

| **Species** | **Smaller** | | | **Larger** | | |
| --- | --- | --- | --- | --- | --- | --- |
|  | **VL < M** | **VL < VH** | **M < VH** | **VL > M** | **VL > VH** | **M > VH** |
| *Lutjanus fulvus* | 0.28 | 0.93 | 0.96 | 0.72 | 0.07 | 0.04 |
| *Cephalopholis urodeta* | 0.00 | 0.01 | 0.87 | 1.00 | 0.99 | 0.13 |
| *Lutjanus bohar* | 0.00 | 0.37 | 0.98 | 1.00 | 0.63 | 0.02 |
| *Cephalopholis argus* | 0.00 | 0.13 | 0.89 | 1.00 | 0.87 | 0.11 |
| *Caranx melampygus* | 0.30 | NA | NA | 0.70 | NA | NA |
| *Aphareus furca* | 0.93 | 0.02 | 0.00 | 0.07 | 0.98 | 1.00 |

Note: “VL < M” quantifies the probability that the SEA-B for *Very Low* disturbance sites is smaller than the SEA-B for *Medium* disturbance sites.

**Table S5.** Comparisons of isotopic niche overlap among human disturbance levels. Mean species-specific posterior probabilities (%) that an individual from one disturbance level (row) would occur within the 95% isotopic niche region of another disturbance level (column). Highlighted cells identify proportion overlap ≥ 50% (light) or ≥ 75% (dark). VL = Very Low, M = Medium, VH = Very High.

| **Species** | **n** | **Disturbance Level** | **VL** | **M** | **VH** | **Species Average** |
| --- | --- | --- | --- | --- | --- | --- |
| *Lutjanus fulvus* | 49 | VL | – | 63.3 | 87.5 |  |
|  |  | M | 70.8 | – | 70.0 |  |
|  |  | VH | 69.4 | 50.1 | – | 68.5 |
| *Cephalopholis urodeta* | 64 | VL | – | 37.6 | 64.3 |  |
|  |  | M | 89.8 | – | 90.5 |  |
|  |  | VH | 93.5 | 71.1 | – | 74.5 |
| *Lutjanus bohar* | 24 | VL | – | 21.9 | 47.3 |  |
|  |  | M | 91.2 | – | 71.9 |  |
|  |  | VH | 85.3 | 26.0 | – | 57.3 |
| *Cephalopholis argus* | 68 | VL | – | 57.0 | 53.6 |  |
|  |  | M | 95.0 | – | 74.6 |  |
|  |  | VH | 87.6 | 61.7 | – | 71.6 |
| *Caranx melampygus* | 11 | VL | – | 38.3 | NA |  |
|  |  | M | 69.5 | – | NA |  |
|  |  | VH | NA | NA | – | 53.9 |
| *Aphareus furca* | 16 | VL | – | 24.8 | 1.6 |  |
|  |  | M | 15.5 | – | 6.5 |  |
|  |  | VH | 32.9 | 93.0 | – | 29.1 |

**Table S6.** Linear discriminant coefficients for the LDA model using δ^13^C values of six essential AAs (Ile, Leu, Ly, Phe, Thr, Val) for reef fish and carbon source end-members.

| **Amino Acid** | **LD_1_** | **LD_2_** | **LD_3_** |
| --- | --- | --- | --- |
| Isoleucine (Ile) | 1.509 | 0.117 | 0.140 |
| Leucine (Leu) | -1.645 | 1.054 | -0.111 |
| Lysine (Lys) | 0.538 | 0.025 | -0.032 |
| Phenylalanine (Phe) | -0.510 | 0.759 | -0.766 |
| Threonine (Thr) | 0.718 | -0.500 | 0.282 |
| Valine (Val) | -0.055 | -0.842 | 1.311 |
| Proportion of trace | 0.835 | 0.139 | 0.026 |

**Table S7.** Correlations between pairs of carbon sources from species-specific Bayesian Stable Isotope Mixing Models. Higher correlations among sources increase marginal uncertainty in proportion estimates (Phillips et al., 2014). Correlations > |0.60| are bolded.

| **Species** | **Variable** | **Coral** | **Detritus** | **Epilithic Algal Matrix** | **Plankton** |
| --- | --- | --- | --- | --- | --- |
| *Lutjanus fulvus* | Coral | 1.00 |  |  |  |
| (n = 15) | Detritus | **–0.87** | 1.00 |  |  |
|  | Epilithic Algal Matrix | 0.00 | –0.11 | 1.00 |  |
|  | Plankton | –0.13 | –0.35 | –0.05 | 1.00 |
| *Cephalopholis urodeta* | Coral | 1.00 |  |  |  |
| (n = 15) | Detritus | **–0.76** | 1.00 |  |  |
|  | Epilithic Algal Matrix | 0.11 | –0.31 | 1.00 |  |
|  | Plankton | –0.34 | –0.24 | –0.30 | 1.00 |
| *Lutjanus bohar* | Coral | 1.00 |  |  |  |
| (n = 16) | Detritus | –0.52 | 1.00 |  |  |
|  | Epilithic Algal Matrix | 0.14 | –0.33 | 1.00 |  |
|  | Plankton | –0.23 | –0.59 | –0.25 | 1.00 |
| *Cephalopholis argus* | Coral | 1.00 |  |  |  |
| (n = 15) | Detritus | **–0.69** | 1.00 |  |  |
|  | Epilithic Algal Matrix | 0.32 | –0.36 | 1.00 |  |
|  | Plankton | 0.21 | **–0.74** | –0.26 | 1.00 |
| *Caranx melampygus* | Coral | 1.00 |  |  |  |
| (n = 10) | Detritus | –0.48 | 1.00 |  |  |
|  | Epilithic Algal Matrix | 0.09 | –0.24 | 1.00 |  |
|  | Plankton | –0.25 | **–0.61** | –0.31 | 1.00 |
| *Aphareus furca* | Coral | 1.00 |  |  |  |
| (n = 13) | Detritus | **–0.62** | 1.00 |  |  |
|  | Epilithic Algal Matrix | 0.06 | –0.38 | 1.00 |  |
|  | Plankton | –0.41 | –0.31 | –0.22 | 1.00 |

**References**

Baum, J. K., Claar, D. C., Tietjen, K. L., Magel, J. M. T., Maucieri, D. G., Cobb, K. M., & McDevitt-Irwin, J. M. (2023). Transformation of coral communities subjected to an unprecedented heatwave is modulated by local disturbance. *Science Advances*, *9*(14), eabq5615. https://doi.org/10.1126/sciadv.abq5615

Besser, A. C., Elliott Smith, E. A., & Newsome, S. D. (2022). Assessing the potential of amino acid δ^13^C and δ^15^N analysis in terrestrial and freshwater ecosystems. *Journal of Ecology*, *110*(4), 935–950.

Bradley, C. J., Wallsgrove, N. J., Choy, C. A., Drazen, J. C., Hetherington, E. D., Hoen, D. K., & Popp, B. N. (2015). Trophic position estimates of marine teleosts using amino acid compound specific isotopic analysis: Stable isotope-derived trophic positions of teleosts. *Limnology and Oceanography: Methods*, *13*(9), 476–493. https://doi.org/10.1002/lom3.10041

Burnham, K. P., & Anderson, D. R. (2002). *Model selection and multimodel inference: A practical information-theoretic approach* (2nd ed). Springer.

Chikaraishi, Y., Ogawa, N. O., Kashiyama, Y., Takano, Y., Suga, H., Tomitani, A., Miyashita, H., Kitazato, H., & Ohkouchi, N. (2009). Determination of aquatic food-web structure based on compound-specific nitrogen isotopic composition of amino acids. *Limnology and Oceanography: Methods*, *7*(11), 740–750. https://doi.org/10.4319/lom.2009.7.740

Choat, J., Clements, K., & Robbins, W. (2002). The trophic status of herbivorous fishes on coral reefs: I:Dietary analyses. *Marine Biology*, *140*(3), 613–623. https://doi.org/10.1007/s00227-001-0715-3

Donaldson, T. J. (2002). Habitat association and depth distribution of two sympatric groupers of the genus Cephalopholis (Serranidae: Epinephelinae). *Ichthyological Research*, *49*(2), 191–193. https://doi.org/10.1007/s102280200025

Elliott Smith, E. A., Fox, M. D., Fogel, M. L., & Newsome, S. D. (2022). Amino acid δ^13^C fingerprints of nearshore marine autotrophs are consistent across broad spatiotemporal scales: An intercontinental isotopic dataset and likely biochemical drivers. *Functional Ecology*, *36*(5), 1191–1203. https://doi.org/10.1111/1365-2435.14017

Filous, A., Friedlander, A., Wolfe, B., Stamoulis, K., Scherrer, S., Wong, A., Stone, K., & Sparks, R. (2017). Movement patterns of reef predators in a small isolated marine protected area with implications for resource management. *Marine Biology*, *164*(1), 2. https://doi.org/10.1007/s00227-016-3043-3

Fox, M. D., Elliott Smith, E. A., Smith, J. E., & Newsome, S. D. (2019). Trophic plasticity in a common reef‐building coral: Insights from δ^13^C analysis of essential amino acids. *Functional Ecology*, *33*(11), 2203–2214. https://doi.org/10.1111/1365-2435.13441

Gelman, A., & Rubin, D. B. (1992). Inference from iterative simulation using multiple sequences. *Statistical Science*, *7*(4), 457–472. https://doi.org/10.1214/ss/1177011136

Geweke, J. (1992). *Evaluating the accuracy of sampling-based approaches to the calculation of posterior moments* (J. M. Bernardo, J. O. Berger, A. P. Dawid, & A. F. M. Smith, Eds.). Claredon Press.

Harmelin-Vivien, M. L., & Bouchon-Navaro, Y. (1983). Feeding diets and significance of coral feeding among Chaetodontid fishes in Moorea (French Polynesia). *Coral Reefs*, *2*(2), 119–127. https://doi.org/10.1007/BF02395282

Holland, K. N., Lowe, C. G., & Wetherbee, B. M. (1996). Movements and dispersal patterns of blue trevally (*Caranx melampygus*) in a fisheries conservation zone. *Fisheries Research*.

Jackson, A. L., Inger, R., Parnell, A. C., & Bearhop, S. (2011). Comparing isotopic niche widths among and within communities: SIBER – Stable Isotope Bayesian Ellipses in R. *Journal of Animal Ecology*, *80*(3), 595–602. https://doi.org/10.1111/j.1365-2656.2011.01806.x

Kindinger, T. L., Adam, T. C., Baum, J. K., Dimoff, S. A., Hoey, A. S., & Williams, I. D. (2024). Herbivory through the lens of ecological processes across Pacific coral reefs. *Ecosphere*, *15*(2), e4791. https://doi.org/10.1002/ecs2.4791

Lindholm, J., Kaufman, L., Miller, S., Wagschal, A., & Newville, M. (2005). Movement of yellowtail snapper (*Ocyurus chrysurus* Block 1790) and black grouper (*Mycteroperca bonaci* Poey 1860) in the northern Florida Keys National Marine Sanctuary as determined by acoustic telemetry (Marine Sanctuaries Conservation Series MSD-05-4.; p. 17). U.S. Department of Commerce, National Oceanic and Atmospheric Administration, Marine Sanctuaries Division.

Liu, M., & Sadovy, Y. (2005). Habitat Association and Social Structure of the Chocolate Hind, Cephalopholis boenak (Pisces: Serranidae: Epinephelinae), at Ping Chau Island, Northeastern Hong Kong Waters. *Environmental Biology of Fishes*, *74*(1), 9–18. https://doi.org/10.1007/s10641-005-2258-9

Luo, J., Serafy, J., Sponaugle, S., Teare, P., & Kieckbusch, D. (2009). Movement of gray snapper Lutjanus griseus among subtropical seagrass, mangrove, and coral reef habitats. *Marine Ecology Progress Series*, *380*, 255–269. https://doi.org/10.3354/meps07911

Lysy, M., Stasko, A., & Swanson, H. (2021). nicheROVER: Niche Region and Niche Overlap Metrics for Multidimensional Ecological Niches. *R Package Version 1.1.0*. https://CRAN.R-project.org/package=nicheROVER

Magel, J. M. T., Dimoff, S. A., & Baum, J. K. (2020). Direct and indirect effects of climate change‐amplified pulse heat stress events on coral reef fish communities. *Ecological Applications*, *30*(6), e02124. https://doi.org/10.1002/eap.2124

Manlick, P. J., & Newsome, S. D. (2022). Stable isotope fingerprinting traces essential amino acid assimilation and multichannel feeding in a vertebrate consumer. *Methods in Ecology and Evolution*, *13*(8), 1819–1830. https://doi.org/10.1111/2041-210X.13903

McMahon, K. W., Berumen, M. L., & Thorrold, S. R. (2012). Linking habitat mosaics and connectivity in a coral reef seascape. Proceedings of the National Academy of Sciences, 109(38), 15372–15376. https://doi.org/10.1073/pnas.1206378109

McMahon, K. W., Fogel, M. L., Elsdon, T. S., & Thorrold, S. R. (2010). Carbon isotope fractionation of amino acids in fish muscle reflects biosynthesis and isotopic routing from dietary protein. *Journal of Animal Ecology*, *79*(5), 1132–1141. https://doi.org/10.1111/j.1365-2656.2010.01722.x

McMahon, K. W., Thorrold, S. R., Houghton, L. A., & Berumen, M. L. (2016). Tracing carbon flow through coral reef food webs using a compound-specific stable isotope approach. *Oecologia*, *180*(3), 809–821. https://doi.org/10.1007/s00442-015-3475-3

Meyer, C. G., Holland, K. N., Wetherbee, B. M., & Lowe, C. G. (2001). Diet, resource partitioning and gear vulnerability of Hawaiian jacks captured in fishing tournaments. *Fisheries Research*, *53*(2), 105–113. https://doi.org/10.1016/S0165-7836(00)00285-X

Meyer, C. G., & Honebrink, R. R. (2005). Transintestinal Expulsion of Surgically Implanted Dummy Transmitters by Bluefin Trevally—Implications for Long‐Term Movement Studies. *Transactions of the American Fisheries Society*, *134*(3), 602–606. https://doi.org/10.1577/T04-082.1

Morate, O. (2016). *2015 Population and Housing Census. Volume 1: Management Report and Basic Tables* (p. 197). National Statistics Office, Ministry of Finance.

Mundy, B. C. (2005). Checklist of the fishes of the Hawaiian Archipelago. *Bishop Museum Bulletin in Zoology*, *6*, 1–704.

Myers, R. F. (1999). *Micronesian reef fishes: A comprehensive guide to the coral reef fishes of Micronesia* (3rd ed.). Coral Graphics.

Nakamura, Y., Horinouchi, M., Shibuno, T., Tanaka, Y., Miyajima, T., Koike, I., Kurokura, H., & Sano, M. (2008). Evidence of ontogenetic migration from mangroves to coral reefs by black-tail snapper Lutjanus fulvus: Stable isotope approach. Marine Ecology Progress Series, 355, 257–266. https://doi.org/10.3354/meps07234

Nanami, A. (2021). Spatial distribution of parrotfishes and groupers in an Okinawan coral reef: Size-related associations in relation to habitat characteristics. *PeerJ*, *9*, e12134. https://doi.org/10.7717/peerj.12134

Newsome, S. D., Martinez del Rio, C., Bearhop, S., & Phillips, D. L. (2007). A niche for isotopic ecology. *Frontiers in Ecology and the Environment*, *5*(8), 429–436. https://doi.org/10.1890/060150.1

Phillips, D. L., Inger, R., Bearhop, S., Jackson, A. L., Moore, J. W., Parnell, A. C., Semmens, B. X., & Ward, E. J. (2014). Best practices for use of stable isotope mixing models in food-web studies. *Canadian Journal of Zoology*, *92*(10), 823–835. https://doi.org/10.1139/cjz-2014-0127

Popple, I. D., & Hunte, W. (2005). Movement patterns of *Cephalopholis cruentata* in a marine reserve in St Lucia, W.I., obtained from ultrasonic telemetry. *Journal of Fish Biology*, *67*(4), 981–992. https://doi.org/10.1111/j.0022-1112.2005.00797.x

R Core Team. (2021). R: A language and environment for statistical computing. *R Foundation for Statistical Computing, Vienna, Austria*.

Ramirez, M. D., Besser, A. C., Newsome, S. D., & McMahon, K. W. (2021). Meta‐analysis of primary producer amino acid δ^15^N values and their influence on trophic position estimation. *Methods in Ecology and Evolution*, *12*, 1750–1767. https://doi.org/10.1111/2041-210X.13678

Randall, J. E. (2005). *Reef and Shore Fishes of the South Pacific: New Caledonia to Tahiti and the Pitcairn Islands*. University of Hawai’i Press.

Rowe, A. G., Iken, K., Blanchard, A. L., O’Briend, D. M., Døving Osvik, R., Uradnikova, M., & Wooller, M. J. (2019). Sources of primary production to Arctic bivalves identified using amino acid stable carbon isotope fingerprinting. *Isotopes in Environmental and Health Studies*, *55*(4), 366–384.

RStudio Team. (2022). RStudio: Integrated Development Environment for R. *RStudio, PBC, Boston, MA*. http://www.rstudio.com/

Shpigel, M., & Fishelson, L. (1989). Habitat partitioning between species of the genus Cephalopholis (Pisces, Serranidae) across the fringing reef of the Gulf of Aqaba (Red Sea). *Marine Ecology Progress Series*, *58*, 17–22. https://doi.org/10.3354/meps058017

Shpigel, M., & Fishelson, L. (1991). Territoriality and associated behaviour in three species of the genus *Cephalopholis* (Pisces: Serranidae) in the Gulf of Aqaba, Red Sea. *Journal of Fish Biology*, *38*(6), 887–896. https://doi.org/10.1111/j.1095-8649.1991.tb03628.x

Silfer, J. A., Engel, M. H., Macko, S. A., & Jumeau, E. J. (1991). Stable carbon isotope analysis of amino acid enantiomers by conventional isotope ratio mass spectrometry and combined gas chromatography/isotope ratio mass spectrometry. *Analytical Chemistry*, *63*(4), 370–374. https://doi.org/10.1021/ac00004a014

Skinner, C., Mill, A. C., Fox, M. D., Newman, S. P., Zhu, Y., Kuhl, A., & Polunin, N. V. C. (2021). Offshore pelagic subsidies dominate carbon inputs to coral reef predators. *Science Advances*, *7*(8), eabf3792. https://doi.org/10.1126/sciadv.abf3792

Spiess, A.-N. (2018). propagate: Propagation of uncertainty. *R Package Version 1.0-6*. https://CRAN.R-project.org/package=propagate

Stock, B. C., Jackson, A. L., Ward, E. J., Parnell, A. C., Phillips, D. L., & Semmens, B. X. (2018). Analyzing mixing systems using a new generation of Bayesian tracer mixing models. *PeerJ*, *6*, e5096. https://doi.org/10.7717/peerj.5096

Sudekum, A. E., Parrish, J. D., Radtke, R. L., & Ralston, S. (1991). Life History and Ecology of Large Jacks in Undisturbed, Shallow, Oceanic Communities. *Fishery Bulletin*, *89*, 493–513.

Ticzon, V. S., Mumby, P. J., Samaniego, B. R., Bejarano-Chavarro, S., & David, L. T. (2012). Microhabitat use of juvenile coral reef fish in Palau. *Environmental Biology of Fishes*, *95*(3), 355–370. https://doi.org/10.1007/s10641-012-0010-9

Tietbohl, M. D. (2016). *Assessing the functional diversity of herbivorous reef fishes using a compound-specific stable isotope approach* [Master’s Thesis]. King Abdullah University of Science and Technology.

Topping, D., & Szedlmayer, S. (2011). Site fidelity, residence time and movements of red snapper *Lutjanus campechanus* estimated with long-term acoustic monitoring. *Marine Ecology Progress Series*, *437*, 183–200. https://doi.org/10.3354/meps09293

Walsh, S. M. (2011). Ecosystem-scale effects of nutrients and fishing on coral reefs. *Journal of Marine Biology*, *2011*, 1–13. https://doi.org/10.1155/2011/187248

Watson, M. S., Claar, D. C., & Baum, J. K. (2016). Subsistence in isolation: Fishing dependence and perceptions of change on Kiritimati, the world’s largest atoll. *Ocean & Coastal Management*, *123*, 1–8. https://doi.org/10.1016/j.ocecoaman.2016.01.012

Whiteman, J. P., Elliott Smith, E. A., Besser, A. C., & Newsome, S. D. (2019). A Guide to Using Compound-Specific Stable Isotope Analysis to Study the Fates of Molecules in Organisms and Ecosystems. *Diversity*, *11*(1), 8. https://doi.org/10.3390/d11010008

Wilson, S., & Bellwood, D. (1997). Cryptic dietary components of territorial damselfishes (Pomacentridae, Labroidei). *Marine Ecology Progress Series*, *153*, 299–310. https://doi.org/10.3354/meps153299

Yarnes, C. T., & Herszage, J. (2017). The relative influence of derivatization and normalization procedures on the compound-specific stable isotope analysis of nitrogen in amino acids: Reproducibility of ^15^ N in amino acids for GC/C/IRMS. *Rapid Communications in Mass Spectrometry*, *31*(8), 693–704. https://doi.org/10.1002/rcm.7832
